# Adherence to the EAT‐Lancet Healthy Reference Diet in Relation to Risk of Cardiovascular Events and Environmental Impact: Results From the EPIC‐NL Cohort

**DOI:** 10.1161/JAHA.122.026318

**Published:** 2023-04-17

**Authors:** Chiara Colizzi, Marjolein C. Harbers, Reina E. Vellinga, W. M. Monique Verschuren, Jolanda M. A. Boer, Sander Biesbroek, Elisabeth H. M. Temme, Yvonne T. van der Schouw

**Affiliations:** ^1^ Julius Center for Health Sciences and Primary Care University Medical Center Utrecht, Utrecht University Utrecht The Netherlands; ^2^ Centre for Nutrition, Prevention and Health Services National Institute for Public Health and the Environment (RIVM) Bilthoven The Netherlands; ^3^ Department of Agrotechnology and Food Science Wageningen University & Research Wageningen The Netherlands

**Keywords:** EAT‐Lancet, healthy reference diet, planetary health, sustainability, Cardiovascular Disease, Diet and Nutrition, Epidemiology, Primary Prevention, Risk Factors

## Abstract

**Background:**

The Healthy Reference Diet (HRD) was created to formulate dietary guidelines that would be healthy and sustainable. We aimed to construct a diet score measuring adherence to the HRD and to explore its association with cardiovascular events and environmental impact.

**Methods and Results:**

We included 35 496 participants from the population‐based EPIC‐NL (European Prospective Investigation into Cancer and Nutrition‐Netherlands) study. HRD scores were calculated using data from food frequency questionnaires (0–140). Data on morbidity and mortality were retrieved through linkage with national and death registries. Data on environmental impact indicators were obtained from life cycle assessments. Associations between adherence to the HRD and cardiovascular events were estimated with Cox proportional hazard models. Linear regression analysis was conducted for the adherence to the HRD and each environmental indicator. High adherence to the HRD was associated with 14%, 12%, and 11% lower risks of cardiovascular disease (hazard ratio [HR]_Q4vsQ1_, 0.86 [95% CI, 0.78–0.94]), coronary heart disease (HR_Q4vsQ1_, 0.88 [95% CI, 0.78–1.00]), and total stroke (HR_Q4vsQ1_, 0.89 [95% CI, 0.72–1.10]), respectively. High HRD adherence was associated with 2.4% (95% CI, −5.0 to 0.2) lower greenhouse gas emissions, 3.9% (95% CI, −5.2 to −2.6) less land use, 0.5% (95% CI, −2.6 to 1.6), less freshwater eutrophication, 3.3% (95% CI, −5.8 to −0.8), less marine eutrophication, 7.7% (95% CI, −10.8 to −4.6), less terrestrial acidification, and 32.1 % (95% CI, 28.5–35.7) higher blue water use.

**Conclusions:**

High adherence to the HRD was associated with lower risk of cardiovascular disease, coronary heart disease, and modestly lower levels of most environmental indicators but a higher level of blue water use.

**Nonstandard Abbreviations and Acronyms**
EPIC: European Prospective Investigation into Cancer and Nutrition
FFQ: food frequency questionnaire
GHGE: greenhouse‐gas emissions
HDI: Healthy Diet Indicator
HRD: Healthy Reference Diet
HRDea: Healthy Reference Diet, energy adjusted
IPTWs: inverse probability of treatment weights
LCA: life cycle assessments
mMED: modified Mediterranean Diet Score
PDI: plant‐based diet index

**Clinical Perspective**

**What Is New?**

- This is the first study that created a nuanced diet score to measure adherence to the Healthy Reference Diet and empirically assessed the potential cardiovascular benefits and environmental impact of the Healthy Reference Diet in a large prospective cohort study.

**What Are the Clinical Implications?**

- The present study suggests that adhering to the Healthy Reference Diet was associated with lower risk of cardiovascular disease and coronary heart disease but was not associated with lower risk of total stroke, ischemic stroke, and hemorrhagic stroke.
- High adherence to the Healthy Reference Diet, although modestly associated with lower levels of greenhouse‐gas emissions, land use, freshwater eutrophication, marine eutrophication, and terrestrial acidification, was also correlated with high increase in the levels of blue water use.

Diet has a profound impact on human health as well as the environment.^1^ According to the Global Burden of Disease Study 2017, 11 million deaths and 255 million disability‐adjusted life years can be attributed to high sodium intakeand low intake of whole grains and fruit across the world.^2^ Unhealthy diets are considered one of the main risk factors for the development of cardiovascular diseases.^3^ At the same time, current dietary practices are likely to exhaust our planet in light of the expected growth of the world population.^1^ Food production practices account for up to 30% of global greenhouse‐gas emissions (GHGE) and 70% of freshwater use, most of which is intended for meat and dairy production.^4^, ^5^, ^6^ For these reasons, shifting toward healthy and sustainable diets could benefit both public and planetary health. A recent statement from the American Heart Association also suggested that, besides improving heart health and other chronic conditions, heart‐healthy dietary patterns could have a substantially lower environmental impact, compared with diets that prefer animal‐based food production and consumption.^7^

The EAT‐Lancet Commission on Healthy Diets From Sustainable Food Systems is the first large‐scale and coordinated scientific collaboration to provide dietary guidelines on healthy diets within the food production boundaries for the world population.^8^ The commission proposed the Healthy Reference Diet (HRD), which was constructed based on scientifically established targets for healthy diets and fitting within a safe operating space for food systems, for which the Planetary Boundaries framework was used. The diet includes high consumption of fruits and vegetables, whole grains, legumes, nuts, and unsaturated oils; low to moderate consumption of dairy, starchy vegetables, poultry, and fish; and no or low consumption of saturated fats, red meat, and all sweeteners.^8^ As such, the HRD generally emphasizes the intake of plant‐based foods and suggests to limit the intake of animal‐sourced foods and starchy vegetables.

The EAT‐Lancet report projected that 19.0% to 23.6% of premature adult deaths could potentially be avoided by adopting the HRD, while remaining within acceptable environmental boundaries.^8^ However, these projections were based on theoretical models. To date, only 2 studies empirically assessed the association between the HRD and the risk of cardiovascular outcomes, with inconclusive findings.^9^, ^10^ Uncertainties in these findings may relate to the dichotomous scoring system that was applied in both studies, which consequently did not allow for large variation in HRD scores.^11^ Thus, evidence on the potential cardiovascular benefits of the HRD coming from prospective cohort studies using a nuanced diet score to measure adherence is currently lacking. Additionally, the environmental impact of the HRD has not been previously assessed empirically. Insight into the cardiovascular and planetary consequences of adhering to the HRD would help to identify win‐win or win‐lose aspects of the HRD.

Therefore, the present study has 3 aims: to construct a refined HRD score allowing for wide variation in adherence to the HRD, to estimate the association of adherence to the HRD with risk of cardiovascular events in a population‐based cohort study, and to estimate the associated environmental impact of the HRD using a wide range of environmental indicators relating to the planetary boundaries in the same cohort study.

## METHODS

The data that support the findings of this study are available from the corresponding author upon reasonable request.

### Study Population

We used data from the Dutch contribution to the EPIC‐NL (European Prospective Investigation into Cancer and Nutrition‐Netherlands).^12^ The EPIC study was designed to study the associations between diet, lifestyle, and the incidence of cancer and other chronic conditions.^12^ The EPIC‐NL cohort combines the MORGEN cohort (n=22 654) and the Prospect cohort (n=17 357), resulting in a total of 40 011 participants. The MORGEN cohort included both men and women, aged 20 to 64 years, from 3 Dutch cities (Amsterdam, Doetinchem, and Maastricht), recruited between 1993 and 1997. The Prospect cohort included women participating in a breast screening program, aged 49 to 70 years, recruited between 1993 and 1995 from Utrecht and its vicinity. At baseline, participants completed a general questionnaire and a validated semi‐quantitative food frequency questionnaire (FFQ). During a physical examination, a nonfasting blood sample was taken, aliquoted, and stored for future research. The EPIC‐NL study was conducted according to the guidelines in the Declaration of Helsinki, and all procedures involving the participants were approved by the institutional review board of the University Medical Center Utrecht (Prospect‐EPIC) and the medical ethical committee of TNO Nutrition and Food Research (MORGEN‐EPIC). All participants provided written informed consent.

For the current study, we excluded participants who withheld permission for linkage with national disease and death registries (n=100), withdrew informed consent during follow‐up (n=1), were missing outcome data (n=1673), were prevalent cases of the outcomes of interest (n=536), had missing dietary intake data (n=218), had implausible energy intake (defined as those in the lowest and highest 0.5% of the ratio of energy intake over basal metabolic rate; n=400), and were missing data on covariates (n=1587), leaving 35 496 individuals for analysis (Figure S1).

### Calculation of the HRD Adherence Score

The FFQ enables assessment of the consumption of 178 food items in the year before enrollment.^12^, ^13^ For some food items, questions were accompanied by images of the food in different portion sizes to assist in portion size estimation. Frequency of consumption was estimated in times per day, week, month, year, or never. Average food intake (g/d) was calculated by multiplying the consumption frequency with the consumed amounts, and nutrient intakes were calculated using the Dutch food composition table of 1996.^14^

To assess adherence to the HRD, an HRD score was constructed. To calculate the adherence scores, the dietary recommendations from the EAT‐Lancet report were prorated based on the individuals' observed intake and recalculated on the basis of 2000 kcal/day for women, in line with the recommended energy intake proposed by the Dutch dietary guidelines (Table S1 and Table S2). Participants were assigned proportional scores ranging from 0 to 10 for each of the 14 dietary recommendations in the HRD (as proposed by EAT‐Lancet) that were then summed, resulting in a score ranging between 0 (no adherence) and 140 (complete adherence). Each food group in the HRD score was categorized into 1 of the following scoring components adapted from Looman et al^15^: adequacy, moderation, optimum, or ratio. The allocation of scoring components to the dietary recommendations in the HRD was informed by literature investigating the associations of those food groups with chronic disease.^16^, ^17^, ^18^, ^19^, ^20^, ^21^, ^22^, ^23^, ^24^, ^25^, ^26^, ^27^, ^28^, ^29^, ^30^, ^31^, ^32^, ^33^, ^34^, ^35^, ^36^, ^37^, ^38^

Adequacy components are used to score foods generally considered healthy and for which a high intake is recommended. In the HRD score, foods assigned to this component were whole grains (converted from raw to cooked),^39^ vegetables, fruits, legumes, and soy foods. Participants received 10 points for meeting the recommended intake for these food groups, 0 points for no consumption, and a proportional score for intakes between 0 and the recommended level. Moderation components were used to score foods that could increase the risk of chronic diseases. The moderation component was used to score beef, lamb, pork, and sweeteners. For these foods, 0 points were assigned if the intake was above the reference intake, 10 points were assigned for an intake equal to or lower than the reference intake, and a proportional score was assigned for intakes between 0 and the recommended level.

Optimum components comprise foods that are nutritious yet potentially detrimental if eaten in large quantities on a daily basis. The optimum component was used to score the following food groups: potatoes, dairy, chicken, eggs, fish, and nuts. For these foods, participants with intakes within the required optimum intake range would receive 10 points, whereas those with intakes lower or higher than the optimum would be scored proportionally and symmetrically from 0 to 10 and from 10 to 0. Participants with no consumption received 0 points. Finally, a ratio component was used to describe the added fats food group. For the added fats, no consumption of unsaturated fats or an unsaturated to saturated fats ratio lower than 0.6 was assigned 0 points, whereas no consumption of saturated fats or an unsaturated to saturated fats ratio higher than 13 was assigned 10 points. Ratios in between were scored proportionally. Cutoffs and threshold values for the ratio component were derived from the 15th percentile and 85th percentile of the intake distribution of the Dutch reference population, as described in Looman et al.^15^ Finally, the calculated HRD score was adjusted for energy intake (HRDea score) using the energy‐adjusted nutrient residual model to remove the variance in dietary intake related to total energy intake.^40^

### Outcomes Ascertainment

Morbidity data were obtained through linkage with disease registries. Linkage was performed based on date of birth, sex, postal code, and general practitioner with a validated probabilistic method.^41^ Information on vital status was obtained through linkage with the Dutch municipal registry. For deceased participants, information on the causes of death was ascertained through linkage with the Causes of Death Registry of the Central Bureau of Statistics.

Total cardiovascular events included both fatal and nonfatal cases of total cardiovascular disease (CVD), based on hospital discharged diagnoses and causes of death. Besides total CVD, we separately analyzed coronary heart disease (CHD), and total, ischemic, and hemorrhagic stroke. Hospitalization for all outcomes was based on the principal diagnoses (*International Classification of Diseases, Tenth Revision* [*ICD‐10*] codes: I20–I26, I46, R96, G45, I50, I60–I67, I69, I70–I74).

Death from each cardiovascular outcome was based on both primary and secondary causes of death. A primary cause of death was defined as death due to a cardiovascular event, and a secondary cause of death was defined as death due to complications of the primary cause, or another disease that could have led to death. All participants were followed until cardiovascular event, death, emigration, or end of follow‐up, whichever came first. Follow‐up for cardiovascular events was complete until December 31, 2010.

### Environmental Impact Assessment

In the present study, we evaluate the effects of the HRD using 6 indicators: GHGE (kg carbon dioxide equivalent per day); land use (m^2^ per year); blue water use (m^3^ per day), which refers to irrigation water; freshwater eutrophication (kg phosphate equivalent per day) and marine eutrophication (kg nitrogen equivalent per day), which define the process by which bodies of water become enriched with excessive nutrients such as nitrogen and phosphorus^42^; and terrestrial acidification (kg sulfur dioxide equivalent per day), which refers to the process by which chemicals in acidifying forms enter the soil, leading to biodiversity loss.^43^

This wide range of environmental indicators was chosen because they provide a holistic assessment of the environmental impact of the HRD per capita. The “planetary boundaries” within the planetary boundaries framework provide the safe operating space for the Earth's biophysical subsystems and or processes^44^ and also underlie the EAT‐Lancet's commission's environmental impact assessments. Within the planetary boundaries framework, the main environmental systems and processes that are affected by food production are climate change, biodiversity loss, land system change, freshwater use, and nitrogen and phosphorus flows.^8^ Within this framework, the state of these systems is further defined by so‐called control variables. As the main environmental systems are interlinked and interdependent, most control variables relate to multiple environmental systems. For example, GHGE is an indicator of biodiversity loss and climate change; land use is an indicator of biodiversity loss and land system change; blue water use is an indicator of biodiversity loss and freshwater use; eutrophication (eg, through application of fertilizer) is an indicator of nitrogen and phosphorus cycles, biodiversity loss and climate change, and terrestrial acidification is an indicator of biodiversity loss.^8^, ^44^, ^45^

The associated environmental impacts of the 178 foods and beverages were assessed using the most recent life cycle assessments (LCA) data from the Dutch LCA Food database.^46^ This database is established by the National Institute for Public Health and the Environment and contains information on the environmental impact for approximately 250 Dutch foods and beverages. A full description of the data and assumptions can be found elsewhere.^47^, ^48^ In short, the LCAs had an attributional approach and hierarchical perspective. System boundaries were from cradle to plate, including primary production, processing, primary packaging, distribution, retail, supermarket, storage, preparation by the consumer (eg, cooking), and incineration of packaging waste. Transport between all phases, except from retail to the consumer, was included. Economic allocation (based on economic value) was applied for all food items, except for milk, where physical allocation (based on product mass) was used. In order to estimate daily environmental impact, LCA data from the Dutch LCA Food database, referred to as primary data, were linked via Dutch Food Composition Database codes to FFQ items. When no primary LCA data were available, LCA data were imputed based on the value of on an adjacent/similar food item for which LCA data were available. Extrapolations were based on the ingredients of the foods and similarity in the variety of foods and of the production system.^49^

### Ascertainment of Covariates

Details on data collection on covariates are described elsewhere.^12^ In short, for age, sex, educational level, smoking status and history, physical activity, and medication use data from the baseline general questionnaire were used. Education was categorized into low (lower vocational training and primary school), moderate (secondary school and intermediate vocational training), and high educational (higher vocational training and university) levels. Smoking status was categorized into never smoker, former smoker, or current smoker. Alcohol intake was assessed from the FFQ and measured in grams/day. Physical activity was categorized into inactive, moderately inactive, moderately active, and active, according to the Cambridge Physical Activity Index.^50^ Total energy intake was also derived from the FFQ and expressed in kcal/day.

The baseline physical examination provided data on body weight and height, blood pressure, and cholesterol levels.^13^ Body mass index (BMI) was calculated as height divided by weight squared, and participants were categorized as normal weight for a BMI ≤24.9 kg/m^2^, overweight for a BMI between 25 and 29.9 kg/m^2^, and obese for a BMI ≥30 kg/m^2^. Both systolic and diastolic blood pressure were measured twice in supine position, from which the mean was taken. Blood pressure measurements were performed on the left arm, using a Boso Oscillomat in the MORGEN‐EPIC cohort, and a random zero sphygmomanometer in the Prospect‐EPIC cohort.^12^ Hypertension was defined as use of hypertensive medication and either systolic blood pressure >140 mm Hg or diastolic pressure >90 mm Hg. Serum total cholesterol (mmol/L) was measured using enzymatic methods.^12^

### Statistical Analysis

All baseline characteristics are reported by quartiles of the HRDea score. Normally distributed continuous variables are presented as means with SDs. Continuous variables with a skewed distribution are presented as median with interquartile range. Categorical variables are presented as counts and percentages. A Cox proportional hazard model was used to obtain hazard ratios (HR) and 95% CIs for the association between quartiles of the HRDea score and risk of all cardiovascular outcomes. The lowest quartile was used as reference. The underlying time variable was age from study entry to either diagnosis, death, loss to follow‐up, or end of follow‐up, whichever came first. The proportional hazards assumption was checked using the Schoenfeld test, with no violations observed.

For all cardiovascular outcomes, the analyses present 3 models. Model 1 was adjusted for age and sex. Model 2 was additionally adjusted for educational level, smoking, alcohol consumption, physical activity, and energy intake. Model 3 was additionally adjusted for the cardiovascular risk factors BMI, cholesterol level, and hypertension.

A number of sensitivity analyses were conducted. First, we repeated the analysis of the HRDea score and the health outcomes of interest with the HRDea score modeled as a continuous variable, per 1 SD. To determine whether a specific food group of the HRDea score could be driving the association, the main analyses were repeated using Model 3 but excluding, one at a time, each food group from the HRDea score. Moreover, all analyses were repeated using stabilized inverse probability of treatment weights (IPTWs), to account for any residual confounding.^51^ IPTW uses propensity scores to balance baseline patient characteristics in the exposed and unexposed groups.^51^ To calculate stabilized IPTW, we used logistic regression to calculate the probability of being exposed and then divided the proportion of individuals in each score category by their probability of being exposed.^51^ The weights are then added to the original data to create a pseudo population in which the analyses are repeated. Additionally, stratified analyses were conducted by age, sex, BMI, and educational level, adjusted for the same confounders as Model 3, to test whether the association with health outcomes differed per subgroup. Lastly, to assess the functional relationship between the HRD and the estimates, we ran Cox proportional hazard models using restricted cubic splines. We constructed the splines using 3 knots and the HRDea score median as a reference point.

Furthermore, to compare the performance of the HRDea score in relation to commonly used dietary indices, analyses were repeated using the modified Mediterannean Diet Score (mMED), the Healthy Diet Indicator (HDI), and the Plant‐based Diet Index (PDI) as exposures and cardiovascular events as outcomes. These scores have been found to lower the risk of cardiovascular morbidity and mortality and have been used in other EPIC studies.^52^, ^53^, ^54^, ^55^, ^56^ The mMED, as described by Trichopoulou et al, includes 9 components: fruits, vegetables, legumes, cereals, fish, meat, dairy products, monounsaturated fats, and polyunsaturated fats.^52^ The score can range from 0 (minimal adherence) to 9 (maximal adherence).^52^ The HDI describes the World Health Organization dietary guidelines and includes 7 components scored either 0 or 1.^54^, ^55^ The components in the updated HDI are saturated fatty acids, polyunsaturated fatty acids, cholesterol, protein, dietary fiber, fruits and vegetables, and free sugars.^55^ The PDI includes 18 food groups, divided into healthy (whole grains, fruits, vegetables, nuts, legumes, vegetable oils, tea and coffee), less healthy (fruit juices, refined grains, potatoes, sugar‐sweetened beverages, sweets and desserts), and animal foods (animal fat, dairy, eggs, fish or seafood, meat, miscellaneous animal foods). The PDI scores food groups between 0 and 5 by assigning positive scores for plant foods and inverse scores to animal foods.^57^

All foods in the FFQ, expressed in grams/day, had an estimated environmental impact calculated with LCA. We used linear regression models to estimate the association between HRDea score and each environmental indicator. In this linear regression the exposure was the HRDea score and the outcome was the environmental indicator, calculated as the sum of the associated environmental impact of the food groups included in the HRD. The lowest quartile was used as reference. The analyses in Model 1 were adjusted for age, sex, and energy intake, which were informed by literature.^48^, ^58^, ^59^ The percentage of difference between Q4 and Q1 and 95% CIs were calculated using bootstrapping and by dividing the estimated coefficient in Q4 by the mean estimate in Q1. Moreover, to test whether there was a threshold effect between the HRDea score and the environmental indicators, adjusted linear regression models were run using restricted cubic splines, with 3 knots and with HRDea score median as reference.

The *P* value for trend across quartiles was estimated by modeling the median value of each quartile as a continuous variable. Statistical significance was set at a 2‐tailed *P*<0.05. All statistical analyses were carried out using STATA 13.SE (StataCorp LP). Reporting was guided by the Strengthening the Reporting of Observational Studies in Epidemiology recommendations for nutritional epidemiology.^60^

## RESULTS

Table 1 shows the baseline characteristics of the study population across quartiles of the HRDea score. The average score was 73 (SD=10) and ranged between 32 and 116. Participants most adherent to the HRD were more likely to be female, have a normal BMI, be highly educated, have never smoked, and consume fewer calories per day compared with the least adherent.

**Table 1:** Baseline Characteristics of the EPIC‐NL Cohort by Quartiles of the HRDea Score (n=35 496)*

Quartiles of HRDea scores (range)				
	Q1 (32–66) (n=8874)	Q2 (67–73) (n=8874)	Q3 (74–79) (n=8874)	Q4 (80–117) (n=8874)
Sex				
Male	3673 (41)	2702 (30)	1770 (20)	1070 (12)
Female	5201 (59)	6172 (70)	7104 (80)	7804 (88)
Age, y	48 (37, 55)	51 (41, 57)	52 (44, 58)	53 (46, 59)
Body mass index				
Normal weight	3939 (45)	4218 (48)	4197 (48)	4666 (53)
Overweight	3568 (41)	3372 (40)	3454 (40)	3132 (36)
Obesity	1239 (14)	1166 (13)	1093 (13)	953 (11)
Educational level				
Low	5594 (63)	5302 (60)	5070 (57)	4362 (49)
Moderate	2073 (23)	1968 (22)	1893 (21)	1908 (22)
High	1207 (14)	1604 (18)	1911 (22)	2604 (29)
Smoking				
Never	2999 (34)	3325 (38)	3580 (40)	3653 (41)
Former	2345 (26)	2715 (31)	2995 (34)	3237 (37)
Current	3530 (40)	2834 (32)	2299 (26)	1984 (22)
Physical activity				
Inactive	818 (9)	677 (8)	612 (7)	484 (6)
Moderately inactive	2140 (24)	2234 (25)	2231 (25)	2177 (25)
Moderately active	2161 (24)	2310 (26)	2328 (26)	2450 (28)
Active	3755 (42)	3653 (41)	3703 (42)	3763 (42)
Alcohol consumption, g/day	6 (1, 18)	5 (1, 16)	5 (1, 15)	5 (1, 15)
Energy intake, kcal/day	2283 (1888, 2756)	2031 (1712, 2443)	1869 (1579, 2209)	1745 (1479, 2058)
Total cholesterol (mmol/L)	5.4 (4.7, 6.1)	5.5 (4.8, 6.2)	5.6 (4.9, 6.3)	5.6 (4.8, 6.3)
Hypertension				
Yes	3091 (35)	3274 (37)	3363 (38)	3238 (37)
No	5783 (65)	5600 (63)	5511 (62)	5636 (64)
Food consumption, g/day				
Whole grains	21 (2, 85)	50 (7, 108)	72 (16, 128)	97 (48, 135)
Vegetables	89 (67, 117)	99 (75, 129)	106 (82, 137)	126 (95, 166)
Fruit	104 (49, 178)	136 (90, 250)	189 (122, 278)	241 (158, 323)
Potatoes and cassava	144 (106, 185)	107 (68, 158)	77 (50, 112)	60 (39, 81)
Dairy foods†	537 (257, 722)	416 (232, 612)	381 (234, 544)	324 (208, 427)
Legumes‡	24 (15, 36)	27 (17, 40)	29 (19, 42)	34 (23, 46)
Soy	0 (0, 0)	0 (0, 0)	0 (0, 0)	0 (0, 1)
Beef, lamb, and pork	104 (72, 137)	94 (60, 124)	83 (52, 111)	64 (35, 97)
Chicken	10 (5, 17)	10 (4, 16)	9 (4, 16)	8 (3, 15)
Eggs	21 (11, 29)	14 (8, 21)	14 (7, 18)	11 (6, 16)
Fish	7 (3, 14)	7 (3, 14)	8 (3, 15)	8 (3, 16)
Nuts	4 (1, 12)	4 (2, 11)	4 (2, 10)	5 (2, 12)
Unsaturated fats	11 (5, 21)	11 (5, 20)	10 (5, 18)	10 (5, 17)
Saturated fats	34 (22, 50)	29 (18, 43)	25 (15, 38)	23 (13, 35)
Added sugars	195 (119, 308)	180 (110, 273)	171 (102, 253)	161 (94, 233)

EPIC‐NL indicates European Prospective Investigation into Cancer and Nutrition‐Netherlands; HRDea score, energy‐adjusted Healthy Reference Diet score; Q1, first quartile; Q2, second quartile; Q3, third quartile; and Q4, fourth quartile.

* Estimates are presented as counts n and percentages (%) or as medians (p25, p75).

^†^ Including whole milk, derivate equivalents, and cheese.

^‡^ Including beans, lentils, and peas.

### HRDea Score and Cardiovascular Events

During a median follow‐up of 15.1 years, a total of 4153 CVD events occurred. High adherence to the HRD was associated with lower risk of CVD in the fully adjusted model (HR_Q4vsQ1_, 0.86 [95% CI, 0.78–0.94]; Table 2). During a median follow‐up of 15.1 years, a total of 2355 CHD events occurred. High adherence to the HRD was associated with a lower risk of CHD (HR_Q4vsQ1_, 0.88 [95% CI, 0.78–1.00]). During a median follow‐up of 15.3 years, there were 838 cases of total stroke and 478 and 233 cases of ischemic and hemorrhagic stroke, respectively. High adherence to the HRD was not statistically significantly associated with total stroke (HR_Q4vsQ1_, 0.89 [95% CI, 0.72–1.10]), ischemic stroke (HR_Q4vsQ1_, 0.90 [95% CI, 0.68–1.20]), and hemorrhagic stroke (HR_Q4vsQ1_, 0.92 [95% CI, 0.61–1.38]) in the fully adjusted model, although the magnitude of associations was comparable to those of CVD and CHD.

**Table 2:** Hazard Ratios and 95% CIs for the Association Between Quartiles of the HRDea Score and Incidence of CVD, CHD, Total, Ischemic, and Hemorrhagic Stroke (n=35 496)

Quartiles of HRDea scores (range)					
	Q1 (32–66) (n=8874)	Q2 (67–73) (n=8874)	Q3 (74–79) (n=8874)	Q4 (80–117) (n=8874)	*P*‐trend
CVD, n	1100	1140	979	934	
Person‐years	126 401	126 562	127 491	127 148	
Unadjusted model	1.00 [ref]	1.04 (0.95–1.12)	0.88 (0.81–0.96)	0.84 (0.77–0.92)	<0.001
Model 1*	1.00 [ref]	0.93 (0.85–1.01)	0.75 (0.69–0.82)	0.73 (0.67–0.80)	<0.001
Model 2†	1.00 [ref]	0.97 (0.90–1.06)	0.82 (0.75–0.90)	0.83 (0.76–0.91)	<0.001
Model 3‡	1.00 [ref]	0.99 (0.91–1.08)	0.84 (0.76–0.92)	0.86 (0.78–0.94)	<0.001
CHD, n	645	641	560	509	
Person‐years	128 826	128 817	129 274	129 189	
Unadjusted model	1.00 [ref]	0.99 (0.89–1.11)	0.86 (0.77–0.97)	0.79 (0.70–0.88)	<0.001
Model 1*	1.00 [ref]	0.92 (0.83–1.03)	0.79 (0·70–0.88)	0.74 (0.66–0.83)	<0.001
Model 2†	1.00 [ref]	0.97 (0.87–1.09)	0.86 (0.77–0.97)	0.85 (0.75–0.97)	0.004
Model 3‡	1.00 [ref]	0.99 (0.88–1.10)	0.88 (0.78–0.99)	0.88 (0.78–1.00)	0.019
Total stroke, n	197	233	203	205	
Person‐years	131 826	131 678	1 371 762	131 317	
Unadjusted model	1.00 [ref]	1.18 (0.98–1.43)	1.03 (0.85–1.25)	1.05 (0.86–1.27)	0.963
Model 1*	1.00 [ref]	1.01 (0.83–1.22)	0.79 (0.65–0.97)	0.79 (0.64–0.96)	0.004
Model 2†	1.00 [ref]	1.05 (0.86–1.27)	0.85 (0.70–1.05)	0.88 (0.71–1.08)	0.091
Model 3‡	1.00 [ref]	1.04 (0.86–1.27)	0.86 (0.70–1.06)	0.89 (0.72–1.10)	0.120
Ischemic stroke, n	112	118	125	123	
Person‐years	132 182	132 105	131 993	131 693	
Unadjusted model	1.00 [ref]	1.05 (0.81–1.37)	1.12 (0.87–1.44)	1.11 (0.86–1.43)	0.388
Model 1*	1.00 [ref]	0.89 (0.69–1.16)	0.85 (0.66–1.11)	0.83 (0.63–1.07)	0.151
Model 2†	1.00 [ref]	0.92 (0.71–1.19)	0.91 (0.70–1.19)	0.90 (0.68–1.19)	0.490
Model 3‡	1.00 [ref]	0.92 (0.70–1.19)	0.92 (0.70–1.20)	0.90 (0.68–1.20)	0.509
Hemorrhagic stroke, n	54	76	45	58	
Person‐years	132 606	132 388	132 543	132 091	
Unadjusted model	1.00 [ref]	1.41 (1.00–2.00)	0.83 (0.56–1.24)	1.08 (0.75–1.57)	0.708
Model 1*	1.00 [ref]	1.19 (0.84–1.69)	0.62 (0.42–0.93)	0.77 (0.53–1.13)	0.026
Model 2†	1.00 [ref]	1.26 (0.88–1.80)	0.69 (0.46–1.04)	0.88 (0.59–1.32)	0.163
Model 3‡	1.00 [ref]	1.25 (0.87–1.80)	0.69 (0.45–1.05)	0.92 (0.61–1.38)	0.241

CHD indicates coronary heart disease; CVD, cardiovascular disease; HRDea score, energy‐adjusted healthy reference diet score; *P*‐trend*, P* value for trend; Q1, first quartile; Q2, second quartile; Q3, third quartile; and Q4, fourth quartile.

* Adjusted for age and sex.

^†^ Adjusted for age, sex, educational level, smoking status, alcohol consumption, physical activity, and energy intake.

^‡^ Adjusted for age, sex, educational level, smoking status, alcohol consumption, physical activity, energy intake, body mass index, hypertension, and total cholesterol levels.

### Sensitivity Analyses

The results of the association between the HRDea score continuous and health outcomes yielded a similar interpretation of the findings as for the analyses in quartiles (Table S3).

Excluding each food group one at a time from the HRDea score showed a slight attenuation of the association with CVD when excluding potatoes (HR_Q4vsQ1_, 0.91 [95% CI, 0.83–0.99]; Table S4), CHD (HR_Q4vsQ1_, 0.95 [95% CI, 0.85–1.08]; Table S5), total stroke (HR_Q4vsQ1_, 1.02 [95% CI, 0.84–1.25]), and ischemic stroke (HR_Q4vsQ1_, 1.23 [95% CI, 0.94–1.61]; Table S6 and Table S7). No attenuation of the association was found for hemorrhagic stroke (Table S8).

Applying IPTWs to address residual confounding by included confounders showed stronger inverse associations for CVD (HR_Q4vsQ1_, 0.82 [95% CI, 0.73–0.92]) and for CHD (HR_Q4vsQ1_, 0.86 [95% CI, 0.74–1.00]), as compared with the main analyses (Table S9). Stratification by age, sex, BMI, and educational level did not show any subgroup of special interest (Figure S2 through S6).

We also compared our findings to the mMED, HDI, and PDI and their association with cardiovascular events. The mMED showed a similar association with all outcomes, except for hemorrhagic stroke, for which a significant inverse association was observed (HR_Q4vsQ1_, 0.63 [95% CI, 0.42–0.93]; Table S10). The HDI showed a slightly lower reduction in risk for CVD (HR_Q4vsQ1_, 0.92 [95% CI, 0.83–1.02]) and CHD (HR_Q4vsQ1_, 0.90 [95% CI, 0.78–1.03]) compared with the HRD (Table S11). The PDI showed a weaker association for CVD (HR, 1.00 [95% CI, 0.99–1.00]) and CHD (HR, 1.00 [95% CI, 0.99–1.00]) compared with the HRD, and no association for the stroke outcomes (Table S12). Table S13 also shows the correlation coefficients between the HRD and the mMED, HDI, and PDI, respectively. Lastly, Cox proportional hazard models using restricted cubic splines did not show any deviation from linearity in the relationship between the HRD score and health outcomes (Figure S7).

### HRDea Score and Environmental Impact

Table 3 shows the means of GHGE, land use, blue water use, freshwater eutrophication, marine eutrophication, and terrestrial acidification across quartiles of the HRDea score of the diet reported at time of enrollment (baseline). Participants most adherent to the HRD were more likely to consume diets that were associated with less GHGE, land use, freshwater eutrophication, marine eutrophication, and terrestrial acidification compared with the least adherent. Yet, diets of those most adherent to the HRD have higher blue water use compared with diets of those least adhering to the HRD.

**Table 3:** Descriptive Statistics of the Environmental Impact Indicators by Quartiles of the HRDea Score (n=35 496)*

	Quartiles of HRDea score (range)			
	Q1 (32–66)	Q2 (67–73)	Q3 (74–79)	Q4 (80–116)
	(n=8874)	(n=8874)	(n=8874)	(n=8874)
Greenhouse gases (kg carbon dioxide equivalent)	6.17×10^0^ (1.69×10^0^)	5.65×10^0^ (1.51×10^0^)	5.32×10^0^ (1.36×10^0^)	4.94×10^0^ (1.29×10^0^)
Land use (m^2^ per year)	3.54×10^0^ (9.4×10^−1^)	3.22×10^0^ (8.4×10^−1^)	3.01×10^0^ (7.6×10^−1^)	2.76×10^0^ (7.2×10^−1^)
Blue water use (m^3^ per day)	1.4×10^−1^ (5×10^−2^)	1.5×10^−1^ (5× 10^−2^)	1.6 × 10^−1^ (5 × 10^−2^)	1.7 × 10^−1^ (5 × 10^−2^)
Freshwater eutrophication (g phosphate equivalent)	4.3×10^−4^ (1.2×10^−4^)	4.0×10^−4^ (1.1×10^−4^)	3.7×10^−4^ (9×10^−5^)	3.5×10^−4^ (9×10^−5^)
Marine eutrophication (kg nitrogen equivalent)	1.0×10^−2^ (3×10^−3^)	1.0×10^−2^ (3×10^−3^)	9×10^−3^ (3×10^−3^)	9×10^−3^ (3×10^−3^)
Terrestrial acidification (g sulfur dioxide equivalent)	6.5×10^−2^ (2.0×10^−2^)	5.9×10^−2^ (1.8 × 10^−2^)	5.5×10^−2^ (1.7 × 10^−2^)	5.0×10^−2^ (1.7×10^−2^)

HRDea score indicates energy‐adjusted healthy reference diet score; m^2^ per year, square meters per year; m^3^ per day, cubic meters per day; Q1, first quartile; Q2, second quartile; Q3, third quartile; and Q4, fourth quartile.

* All values are presented as means (SD).

In multivariable adjusted models, high adherence to the HRD was associated with lower GHGE (β=22121.5×10^−1^ kg carbon dioxide equivalent [95% CI, 1.8×10^−1^ to −1.2×10^−1^]), less land use (β=22121.4×10^−1^ m^2^ per year [95% CI, −1.5×10^−1^ to −1.2×10^−1^]), less freshwater eutrophication (β=−2×10^−6^ kg phosphate equivalent [95% CI, −3×10^−6^ to 0×10^0^]), less marine eutrophication (β=−3.3×10^−4^ kg nitrogen equivalent [95% CI, −4.0×10^−4^ to −2.6×10^−4^]), and less terrestrial acidification (β = −5×10^−3^ kg sulfur dioxide equivalent [95% CI, −5×10^−3^ to −4×10^−3^]) and with higher blue water use (β=4.5×10^−2^ [95% CI, 4.3×10^−2^ to 4.6×10^−2^]) when comparing extreme quartiles (Figure). These beta‐coefficients correspond to 2.4% (95% CI, −5.0 to 0.2) lower GHGE, 3.9% (95% CI, −5.2 to −2.6) less land use, 0.5% (95% CI, −2.6 to 1.6) less freshwater eutrophication, 3.3% (95% CI, −5.8 to −0.8) less marine eutrophication, 7.7% (95% CI, −10.8 to −4.6) less terrestrial acidification, and 32.1% (95% CI, 28.5 to 35.7) higher blue water use, when comparing extreme quartiles (Table S14). Linear regression models using restricted cubic splines showed a potential threshold effect for the environmental indicators (Figure S8).

**Figure:**
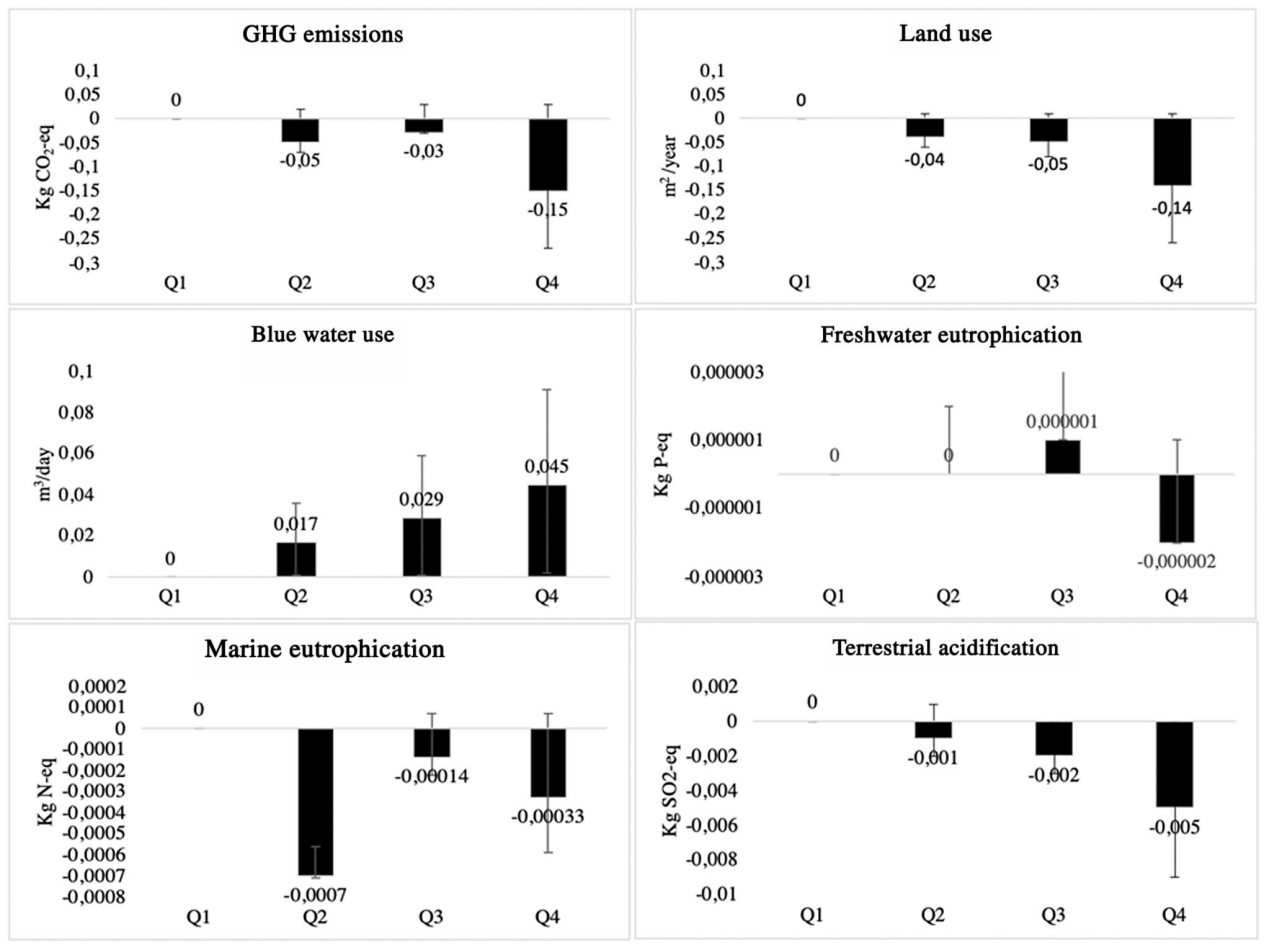
Regression coefficients and 95% CIs for the association between quartiles of the HRDea score and environmental indicators, adjusted for age, sex, and energy intake (n=35 496). GHG indicates global greenhouse gas; HRDea score, energy‐adjusted healthy reference diet score; Kg CO_2_‐eq = kilograms of carbon dioxide equivalent; Kg N‐eq, kilograms of nitrogen equivalent; Kg P‐eq, kilograms of phosphorus equivalent; Kg SO_2_‐eq, kilograms of sulfur dioxide equivalent; m^2^ per year, square meters per year; m^3^ per day, cubic meters per day; Q1, first quartile; Q2, second quartile; Q3, third quartile; and Q4, fourth quartile.

## DISCUSSION

In the present study among 35 496 Dutch adults, we found that higher adherence to the HRD as proposed by the EAT‐Lancet Commission was associated with a 14% lower risk of CVD and a 12% lower risk of CHD. No significant association was found for total stroke, ischemic stroke, and hemorrhagic stroke, although the number of cases was relatively small for stroke subtypes and the magnitude of associations was comparable to those of CVD and CHD. Higher adherence to the HRD was also associated with 3.5% lower GHGE, 2.6% less land use, 0.5% less freshwater eutrophication, 2.5% less marine eutrophication, and 8.1% less terrestrial acidification but with 30.6% higher blue water use.

Before we can interpret our results, we need to address the limitations of the present study. Even though overall the FFQ was considered adequate to assess food intake of the EPIC‐NL population, the validity of vegetable and fish intake was found to be quite poor.^13^ This would suggest possible measurement error in the present study for vegetable and fish intake as well. As misclassification of these food groups is likely to be random given the prospective design of the present study and considering that these food groups are generally associated with lower risk for cardiovascular outcomes, misclassification of the intake of these foods could lead to an attenuation of the associations with the risk of cardiovascular events. Similarly, there may be underestimation of the effects on environmental impact indicators. Moreover, dietary assessment was conducted only at baseline, and dietary intake might have changed during follow‐up. However, a previous study in EPIC‐NL showed dietary changes between baseline and 20‐year follow‐up to be relatively modest.^61^ Nevertheless, although the mean intakes of diet may have not changed substantially, individual variation could still be substantial and lead to attenuation of associations. With regard to our analyses, we need to address the possibility of residual confounding. The data used did not have information on socioeconomic status or occupation, thus, educational level was used as proxy for socioeconomic status. Even though education is one of the factors contributing to socioeconomic differences, it does not capture the whole spectrum of influence of socioeconomic status on diet and health. Therefore, additional information would be needed to account for socioeconomic differences in the study population.

Finally, even though LCA is the most common tool to estimate environmental impacts of foods, several studies have highlighted the limitations of this methodology, such as definition of clear system boundaries, challenges in conducting inventory analysis, and uncertainties in choosing the appropriate environmental impacts.^62^, ^63^, ^64^ The current study used the Dutch LCA Database to calculate environmental indicators. It should be noted that, although the Dutch LCA Database is a comprehensive source of LCA indicators, there is also some uncertainty in the data.^48^ Primary LCA data were available for 242 foods, covering 71% of all foods consumed in the Dutch National Food Consumption Survey.^48^ Furthermore, LCA estimates for the Netherlands will likely not be fully generalizable to other contexts, considering that assumptions and scenarios on which LCA indicators are modeled are country specific.^65^

The main strength of this study is the use of a prospective design, based on a large population cohort, and a long follow‐up period. Moreover, we used proportional scoring from 0 to 10 for each component of the HRDea score, which is likely to capture much of the variability in dietary intake. Additionally, the current study created a nuanced diet score that could be used or adapted by other researchers who wish to study the HRD in other settings. Another strength is the linkage with national registries to ascertain health outcomes, which is considered a valid method to reach near‐complete follow‐up and to reduce possible outcome misclassification.^66^ Furthermore, this study addressed potential residual confounding by conducting sensitivity analyses using IPTW. Applying IPTWs slightly strengthened the associations found between adherence to the HRDea score and CVD and CHD but did not substantially affect the overall interpretation of the results. Finally, the present study included a wide range of environmental indicators, which appeals to the need for an integrated analysis of the core environmental impact dimensions of food systems.^8^

The EAT‐Lancet report leaves some space for definition of the HRD, so that recommendations can be tailored to different populations. Thus, for the construction of the HRDea score, several choices were made in assigning foods to each scoring component, such as the inclusion of dairy and starchy vegetables in the optimum component. Depending on the population and cultural context, some might prefer assigning these food groups to an adherence or moderation component. Additionally, intake recommendations in grams/day from the EAT‐Lancet report were energy adjusted for women, to account for their generally lower energy requirements. Because these choices were mostly based on the baseline characteristics of this study population, they might not be entirely appropriate when replicating this study in a different setting.

Findings from the present study are largely in line with the study from Knuppel et al, which used a simpler score to reflect HRD adherence and found similar inverse associations for CHD risk.^9^ The study by Ibsen et al found a significant association of HRD adherence with subarachnoid stroke, but we cannot exclude that this is a chance finding, given the small number of 115 cases of subarachnoid hemorrhage in total. Our study was in line with the study by Ibsen et al on the null finding for ischemic stroke.^10^

Even though the HRDea score is unique to this study, other studies investigating dietary indices focusing on plant‐based diets show inverse associations with several cardiovascular events.^57^, ^67^ Differences in the magnitude of risk reductions between the present study and available literature are likely related to the scoring methods, the baseline characteristics of the populations, or to residual confounding. This study also compared the HRD to the performance of other commonly used dietary indices, the mMED, HDI, and PDI. Previous studies have estimated the associations between these heart‐healthy dietary patterns and different cardiovascular outcomes.^31^, ^53^, ^68^ In this study, the mMED showed a similar reduction in risk for all cardiovascular outcomes, except for hemorrhagic stroke, for which the association was stronger compared with adherence to the HRD. The HDI, instead, showed a less pronounced or similar reduction in risk for most cardiovascular end points compared with the HRDea score. For the PDI, the associations with CVD, CHD, and stroke outcomes were less pronounced than for other dietary scores or absent.

Further analyses excluding single food groups from the HRDea score did not clearly indicate individual food groups driving the associations. In fact, there was only a slight attenuation in the association with all cardiovascular outcomes when excluding potatoes intake and small attenuation for the risk of total and ischemic stroke when excluding dairy. Overall, this suggests a synergistic effect of various food groups driving the association with cardiovascular and mortality outcomes, rather than food‐specific correlation.

With regard to the environmental impact of the HRD, the indicators used in this study are largely in line with the planetary boundaries framework,^44^, ^45^ which is also applied by the EAT‐Lancet Commission to model the environmental effects of the HRD. Although there was a significant increase in blue water use, the observed percentage reductions for GHGE, land use, freshwater eutrophication, marine eutrophication, and terrestrial acidification in the fully adjusted models seem modest. These findings are in line with findings from the EAT‐Lancet Commission showing that dietary changes alone are not sufficient to stay within most planetary boundaries, except for GHGE, for which a reduction of 49% was observed when comparing current diets with the HRD.^8^ The discrepancy in GHGE reductions between the EAT‐Lancet report and the current study could be due to the fact that in the EPIC‐NL population the maximum HRDea score reached was only 116, whereas complete adherence would yield 140 points. Thus, observed diets may still be suboptimal, and further improvements toward the HRD may have larger effects on environmental impact indicators. For the other environmental indicators, more changes on a societal, organizational, and economic level are needed to have a meaningful change in the environment.^8^, ^69^, ^70^, ^71^, ^72^ These include changes in food waste management, food processing, production, distribution, and transportation.^69^, ^70^, ^71^, ^72^

Adherence to the mMED, HDI, and PDI could not be analyzed in relation to environmental indicators, given that these scores also include nutrients whereas the Dutch LCA Food database is food based.^56^ The Dutch Healthy Diet index 2015—a diet score reflecting adherence to the Dutch national dietary guidelines—has previously been related to environmental sustainability in the EPIC‐NL cohort. In line with our findings, these studies also observed adherence to the Dutch Healthy Diet index 2015 to be associated with lower GHGE and less land use but with higher use of blue water.^56^, ^73^ Indeed, several plant‐based foods—which are emphasized in both the Dutch Healthy Diet index 2015 and the HRD—do have a relatively high blue water use per kg product, such as several fruits and nuts.^74^ Plant‐based foods with a relatively high blue water use are often imported into the Netherlands from areas with a high water scarcity, such as citrus fruits from Spain or almonds from the United States. In order to reduce the blue water footprint of the HRD diet in the Dutch context, it may be recommended to choose locally grown and seasonal fruits and vegetables.^74^ Thus, it is plausible that with small changes in the choice for type of fruits, and when choosing for seasonal and locally grown fruits and vegetables, the high blue water use associated with high HRD adherence could be diminished.

### Conclusions

Current dietary guidelines, including the American Heart Association dietary guidelines, are based on maximization of health benefits. However, it has become evident that other dimensions of dietary patterns, such as sustainability,^75^, ^76^ nutritional adequacy,^77^, ^78^ accessibility,^79^ affordability,^80^ cultural sensitivity, and acceptability^81^, ^82^ of the diet, are of importance as well. The present study showed that taking into account the dimensions of healthy and sustainable diets gives rise to synergies but also trade‐offs. Future dietary guidelines will need to balance all these aspects.

This study provides evidence for an inverse association of adherence to the HRD with CVD and CHD. This study also found that higher adherence to the HRD was associated with some of the environmental indicators included in this research. However, high adherence to the diet was not equally strongly associated with all indicators and was correlated with a high increase in the levels of blue water use.

## Sources of Funding

M.C. Harbers was supported by the Netherlands Cardiovascular Research Initiative, an initiative with support of the Dutch Heart Foundation (CVON2016‐04) and The Netherlands Organisation for Health Research and Development (531003001), in the context of the Supreme Nudge project. Funders had no role in the design of the study and collection, analysis, and interpretation of data and in writing the article, nor have they authority on the decision to submit the article for publication. All authors had full access to all the data in the study and accept responsibility to submit for publication.

## Disclosures

None.

## Supporting information

Data S1
