# Supplementary material for: Adherence to the EAT‐Lancet Healthy Reference Diet in Relation to Risk of Cardiovascular Events and Environmental Impact: Results From the EPIC‐NL Cohort
Source: J Am Heart Assoc. 2023 Apr 17;12(8):e026318. doi: 10.1161/JAHA.122.026318 (PMC10227249; doi:10.1161/JAHA.122.026318)
Supplement: Supplementary file 1 — Data S1 [file JAH3-12-e026318-s001.pdf]

SUPPLEMENTAL MATERIAL

Table S1. Construction of HRD-score, intake recommendations (g/day) and scoring system for men based on 2500 kcal/day.

Food Group	Component type*	HRD recommendation (g/day)	Minimum score (0 points)	Proportional score	Maximum points (10 points)	Proportional score
**Whole Grains**[†]						
Rice, wheat, corn, and other	A	464 (or 60% of total energy)	0 g/d	0-464 g/d	≥464 g/d	
**Vegetables**						
All vegetables[‡]	A	300	0 g/d	0-300 g/d	≥300 g/d	
**Fruits**						
All fruit[§]	A	200	0 g/d	0-200 g/d	≥200 g/d	
**Tubers or starchy vegetables**						
Potatoes and cassava	O	50	0 g/d	0-50 g/d	50-100 g/d	100-150 g/d
**Dairy foods**						
Whole milk or derivative equivalents (e.g., cheese)	O	250	0 g/d	0-250 g/d	250 – 500 g/d	500-750 g/d
**Protein Sources**						
Dry beans, lentils, and peas	A	50	0 g/d	0-50 g/d	≥50 g/d	
Soy foods	A	25	0 g/d	0-25 g/d	≥25 g/d	
Beef, lamb and pork	M	14	≥14 g/d	14 -0 g/d	0 g/d	
Chicken and other poultry	O	29	0 g/d	0-29 g/d	29-58 g/d	58-88 g/d
Eggs	O	13	0 g/d	0-13 g/d	13-25 g/d	25-38 g/d

Fish	O	28	0 g/d	0-28 g/d	28-100 g/d	100-128 g/d
Nuts	O	50	0 g/d	0-50 g/d	50-100 g/d	100-150 g/d
**Added sugars**						
All sweeteners	M	31	≥31 g/d	31-0 g/d	0 g/d	
**Added fats**	R					
Palm oil		6.8	No consumption of		No consumption of	
unsaturated oils		40	unsaturated fats OR		saturated fats OR ratio	
dairy fats included in milk		0	ratio of unsaturated to		of unsaturated to	
lard and tallow		5	saturated fats of ≤0.6 ‖		saturated fats of ≥13 ‖	

*A = adequacy component; O = optimum component; M = moderation component; R = ratio component.

† Reference diet refers to dry, raw weight. Recommendations for whole grains were converted, as described by Dooren et al.[39]

‡ Including dark green vegetables, red and orange vegetables, other vegetables.

§ Excluding fruit juice.

‖ Cut-offs and threshold values were derived from the 15th percentile and 85th percentile of the intake distribution of the Dutch reference population, as described in Looman et al.[15]

Table S2. Construction of HRD-score, intake recommendations (g/day) and scoring system for women based on 2000 kcal/day.

Food Group	Component type*	HRD recommendation (g/day)	Minimum score (0 points)	Proportional score	Maximum points (10 points)	Proportional score
**Whole Grains**†						
Rice, wheat, corn, and other	A	372 (or 60%en)	0 g/d	0-372 g/d	≥372 g/d	
**Vegetables**						
All vegetables‡	A	240	0 g/d	0-240 g/d	≥240 g/d	
**Fruits**						
All fruit§	A	160	0 g/d	0-160 g/d	≥160 g/d	
**Tubers or starchy vegetables**						
Potatoes and cassava	O	40	0 g/d	0-40 g/d	40-80 g/d	80-120 g/d
**Dairy foods**						
Whole milk or derivative equivalents (e.g., cheese)	O	200	0 g/d	0-200 g/d	200 – 400 g/d	400-600 g/d
**Protein Sources**						
Dry beans, lentils, and peas	A	40	0 g/d	0-40 g/d	≥40 g/d	
Soy foods	A	20	0 g/d	0-20 g/d	≥20 g/d	
Beef, lamb and pork	M	12	≥12 g/d	12-0 g/d	0 g/d	
Chicken and other poultry	O	23	0 g/d	0-23 g/d	23-46 g/d	46-69 g/d
Eggs	O	10	0 g/d	0-10 g/d	10-20 g/d	20-30 g/d
Fish	O	22	0 g/d	0-22 g/d	22-80 g/d	80-102 g/d

Nuts	O	40	0 g/d	0-40 g/d	40-80 g/d	80-120 g/d
**Added sugars**						
All sweeteners	M	25	≥25 g/d	25-0 g/d	0 g/d	
**Added fats**	R					
Palm oil		5	No consumption of		No consumption of	
unsaturated oils		32	unsaturated fats OR		unsaturated fats OR	
dairy fats included in milk		0	ratio of unsaturated to		ratio of unsaturated to	
lard and tallow		4	saturated fats of ≤0.5 ‖		saturated fats of ≤0.5 ‖	

*A = adequacy component; O = optimum component; M = moderation component; R = ratio component.
† Reference diet refers to dry, raw weight. Recommendations for whole grains were converted, as described by Dooren et al.[39]
‡ Including dark green vegetables, red and orange vegetables, other vegetables.
§ Excluding fruit juice.
‖ Cut-offs and threshold values were derived from the 15th percentile and 85th percentile of the intake distribution of the Dutch reference population, as described in Looman et al.[15]

Table S3. Hazard ratios (HRs) and 95% confidence intervals (CIs) for the association between HRDea-score (continuous) and incidence of CVD, CHD, total, ischaemic, and haemorrhagic stroke (*n*=35,496).

		HRDea-score		
Outcome		HR	95% CI	*P*-value
CVD, *n*	4,153			
Unadjusted Model		0.92	(0.89 – 0.95)	<0.001
Model 1[a]		0.87	(0.84 – 0.90)	<0.001
Model 2[b]		0.91	(0.88 – 0.95)	<0.001
Model 3[c]		0.93	(0.89 – 0.96)	<0.001
CHD, *n*	2,355			
Unadjusted Model		0.90	(0.86 – 0.93)	<0.001
Model 1[a]		0.87	(0.83 – 0.91)	<0.001
Model 2[b]		0.92	(0.88 – 0.97)	0.001
Model 3[c]		0.94	(0.89 – 0.98)	0.007
Total Stroke, *n*	838			
Unadjusted Model		1.01	(0.94 - 1.08)	0.781
Model 1[a]		0.91	(0.84 – 0.98)	0.013
Model 2[b]		0.95	(0.88 – 1.03)	0.232
Model 3[c]		0.96	(0.88 – 1.04)	0.309
Ischemic stroke, *n*	478			
Unadjusted Model		1.06	(0.97 - 1.17)	0.218
Model 1[a]		0.96	(0.87 - 1.06)	0.372
Model 2[b]		1.00	(0.90 – 1.11)	0.950
Model 3[c]		1.01	(0.90 – 1.12)	0.890
Haemorrhagic stroke, *n*	233			
Unadjusted Model		0.97	(0.85 - 1.10)	0.594
Model 1[a]		0.84	(0.73 – 0.97)	0.018
Model 2[b]		0.89	(0.77 – 1.04)	0.140
Model 3[c]		0.90	(0.77 – 1.05)	0.164

[a] Adjusted for age and sex. [b] Adjusted for age, sex, educational level, smoking status, alcohol consumption, physical activity, and energy intake. [c] Adjusted for age, sex, educational level, smoking status, alcohol consumption, physical activity, energy intake, BMI, hypertension, and total cholesterol levels.

HR = Hazard Ratios
95% CI = 95% Confidence Intervals
HRDea-score = Energy-adjusted Healthy Reference Diet score
CVD = cardiovascular disease
CHD = coronary heart disease

Table S4. Hazard ratios (HRs) and 95% confidence intervals (CIs) for the association between quartiles of the HRDea-score and incidence of cardiovascular disease events, excluding, one at a time, each component of the HRDea-score (*n*=35,496).

	HRDea-score				
	Q1 (32-66) (*n*=8874)	Q2 (67-73) (*n*=8874)	Q3 (74-79) (*n*=8874)	Q4 (80-117) (*n*=8874)	*P*-trend
HR (95% CI)					
HRDea-score recalculated excluding*:					
Whole Grains	1.00 [Reference]	0.95 (0.87, 1.04)	0.85 (0.78, 0.93)	0.85 (0.77, 0.93)	<0.001
Vegetables	1.00 [Reference]	0.99 (0.91, 1.08)	0.83 (0.76, 0.91)	0.86 (0.78, 0.95)	<0.001
Fruit	1.00 [Reference]	0.91 (0.83, 0.99)	0.84 (0.77, 0.92)	0.85 (0.77, 0.94)	<0.001
Potatoes	1.00 [Reference]	0.95 (0.88, 1.03)	0.90 (0.83, 0.98)	0.91 (0.83, 0.99)	0.012
Dairy	1.00 [Reference]	0.96 (0.88, 1.04)	0.87 (0.80, 0.95)	0.83 (0.75, 0.91)	<0.001
Legumes	1.00 [Reference]	0.90 (0.82, 0.98)	0.79 (0.72, 0.87)	0.80 (0.73, 0.89)	<0.001
Soy	1.00 [Reference]	0.98 (0.90, 1.07)	0.86 (0.79, 0.94)	0.83 (0.75, 0.91)	<0.001
Beef, Lamb, Pork	1.00 [Reference]	0.96 (0.88, 1.04)	0.83 (0.76, 0.90)	0.82 (0.74, 0.89)	<0.001
Chicken	1.00 [Reference]	0.97 (0.89, 1.05)	0.83 (0.76, 0.90)	0.83 (0.75, 0.90)	<0.001
Eggs	1.00 [Reference]	0.94 (0.87, 1.02)	0.86 (0.79, 0.94)	0.82 (0.75, 0.90)	<0.001
Fish	1.00 [Reference]	0.97 (0.90, 1.05)	0.82 (0.75, 0.89)	0.83 (0.76, 0.91)	<0.001
Nuts	1.00 [Reference]	0.97 (0.90, 1.05)	0.82 (0.75, 0.89)	0.83 (0.76, 0.91)	<0.001
Sugars	1.00 [Reference]	0.95 (0.88, 1.03)	0.81 (0.74, 0.88)	0.81 (0.74, 0.89)	<0.001
Fats ratio	1.00 [Reference]	0.93 (0.86, 1.01)	0.81 (0.74, 0.88)	0.80 (0.73, 0.88)	<0.001

*Adjusted for: sex, age, educational level, smoking status, alcohol consumption, physical activity, energy intake, BMI, hypertension, and cholesterol levels.

Q1 = first quartile
Q2 = second quartile
Q3 = third quartile
Q4 = fourth quartile
HR = Hazard Ratios
95% CI = 95% Confidence Intervals
p-trend = p-value for trend
HRDea-score = Energy-adjusted Healthy Reference Diet score
CVD = cardiovascular disease

Table S5. **Hazard ratios (HRs) and 95% confidence intervals (CIs) for the association between quartiles of HRDea-score and CHD, excluding, one at a time, each component of the HRD-score (*n*=35,496).**

	**HRDea-score**				
	Q1 (32-66) (*n*=8874)	Q2 (67-73) (*n*=8874)	Q3 (74-79) (*n*=8874)	Q4 (80-117) (*n*=8874)	*P*-trend
HR (95% CI)					
HRD-score recalculated excluding*:					
Whole Grains	1.00 [Reference]	0.94 (0.84 – 1.05)	0.87 (0.77 – 0.98)	0.89 (0.78 – 1.01)	0.034
Vegetables	1.00 [Reference]	1.01 (0.90 – 1.13)	0.89 (0.79 – 1.00)	0.92 (0.81 -1.04)	0.061
Fruit	1.00 [Reference]	0.97 (0.87 – 1.08)	0.87 (0.77 – 0.98)	0.90 (0.79 - 1.02)	0.037
Potatoes	1.00 [Reference]	1.00 (0.89 – 1.12)	0.97 (0.86 - 1.08)	0.95 (0.85 – 1.08)	0.397
Dairy	1.00 [Reference]	0.98 (0.88 – 1.10)	0.91 (0.81 – 1.03)	0.86 (0.75 – 0.98)	0.016
Legumes	1.00 [Reference]	0.94 (0.84 – 1.05)	0.88 (0.78 – 0.99)	0.83 (0.73 – 0.94)	0.002
Soy	1.00 [Reference]	0.97 (0.87 – 1.09)	0.88 (0.78 – 0.99)	0.85 (0.75 – 0.97)	0.006
Beef, Lamb, Pork	1.00 [Reference]	0.97 (0.87 - 1.09)	0.88 (0.78 – 1.00)	0.87 (0.76 – 0.99)	0.013
Chicken	1.00 [Reference]	1.00 (0.89 - 1.11)	0.90 (0.80 – 1.01)	0.89 (0.78 – 1.01)	0.032
Eggs	1.00 [Reference]	0.96 (0.86 – 1.07)	0.88 (0.78 – 0.99)	0.89 (0.78 – 1.01)	0.034
Fish	1.00 [Reference]	0.99 (0.89 – 1.11)	0.88 (0.78 – 0.99)	0.88 (0.77 – 1.00)	0.016
Nuts	1.00 [Reference]	0.99 (0.88 - 1.11)	0.88 (0.78 – 1.00)	0.88 (0.77 – 1.00)	0.017
Sugars	1.00 [Reference]	0.97 (0.86 – 1.08)	0.87 (0.77 – 0.98)	0.85 (0.75 – 0.97)	0.006
Fats ratio	1.00 [Reference]	0.94 (0.84 – 1.05)	0.84 (0.75 – 0.95)	0.86 (0.76 – 0.98)	0.006

* Adjusted for: sex, age, educational level, smoking status, alcohol consumption, physical activity, energy intake, BMI, hypertension, and cholesterol levels.

Q1 = first quartile
Q2 = second quartile
Q3 = third quartile
Q4 = fourth quartile
HR = Hazard Ratios
95% CI = 95% Confidence Intervals
p-trend = p-value for trend
HRDea-score = Energy-adjusted Healthy Reference Diet score
CHD = coronary heart disease

Table S6. Hazard ratios (HRs) and 95% confidence intervals (CIs) for the association between quartiles of the HRDea-score and incidence of total stroke, excluding, one at a time, each component of the HRD-score (*n*=35,496).

	HRDea-score				
	Q1 (32-66) (*n*=8874)	Q2 (67-73) (*n*=8874)	Q3 (74-79) (*n*=8874)	Q4 (80-117) (*n*=8874)	*P*-trend
HR (95% CI)					
HRDea-score recalculated excluding*:					
Whole Grains	1.00 [Reference]	1.00 (0.82, 1.22)	0.93 (0.76, 1.14)	0.88 (0.71, 1.10)	0.202
Vegetables	1.00 [Reference]	0.99 (0.82, 1.21)	0.83 (0.68, 1.02)	0.88 (0.72, 1.09)	0.123
Fruit	1.00 [Reference]	0.88 (0.72, 1.07)	0.85 (0.70, 1.04)	0.85 (0.69, 1.05)	0.129
Potatoes	1.00 [Reference]	1.05 (0.86, 1.28)	0.95 (0.78, 1.17)	1.02 (0.84, 1.25)	0.976
Dairy	1.00 [Reference]	1.07 (0.88, 1.31)	0.95 (0.77, 1.17)	0.90 (0.72, 1.12)	0.228
Legumes	1.00 [Reference]	0.92 (0.76, 1.11)	0.79 (0.64, 0.97)	0.82 (0.67, 1.02)	0.038
Soy	1.00 [Reference]	0.99 (0.81, 1.20)	0.85 (0.69, 1.04)	0.82 (0.66, 1.01)	0.031
Beef, Lamb, Pork	1.00 [Reference]	1.01 (0.83, 1.23)	0.86 (0.70, 1.06)	0.84 (0.68, 1.04)	0.055
Chicken	1.00 [Reference]	1.07 (0.88, 1.30)	0.86 (0.70, 1.06)	0.91 (0.74, 1.13)	0.170
Eggs	1.00 [Reference]	0.94 (0.77, 1.14)	0.94 (0.77, 1.15)	0.81 (0.65, 1.00)	0.060
Fish	1.00 [Reference]	1.04 (0.86, 1.27)	0.86 (0.70, 1.06)	0.89 (0.71, 1.10)	0.117
Nuts	1.00 [Reference]	1.04 (0.86, 1.27)	0.86 (0.70, 1.06)	0.89 (0.72, 1.11)	0.132
Sugars	1.00 [Reference]	1.00 (0.83, 1.22)	0.84 (0.69, 1.04)	0.84 (0.68, 1.04)	0.046
Fats ratio	1.00 [Reference]	0.97 (0.80, 1.18)	0.89 (0.73, 1.09)	0.83 (0.67, 1.03)	0.067

* Adjusted for: sex, age, educational level, smoking status, alcohol consumption, physical activity, energy intake, BMI, hypertension, and cholesterol levels.

Q1 = first quartile
Q2 = second quartile
Q3 = third quartile
Q4 = fourth quartile
HR = Hazard Ratios
95% CI = 95% Confidence Intervals
p-trend = p-value for trend
HRDea-score = Energy-adjusted Healthy Reference Diet score

Table S7. Hazard ratios (HRs) and 95% confidence intervals (CIs) for the association between quartiles of the HRDea-score and incidence of ischaemic stroke, excluding, one at a time, each component of the HRDea-score (*n*=35,496).

	**HRDea-score**				
	Q1 (32-66) (*n*=8874)	Q2 (67-73) (*n*=8874)	Q3 (74-79) (*n*=8874)	Q4 (80-117) (*n*=8874)	*P*-trend
HR (95% CI)					
HRDea-score recalculated excluding*:					
Whole Grains	1.00 [Reference]	0.91 (0.69, 1.19)	1.00 (0.76, 1.30)	0.87 (0.66, 1.16)	0.471
Vegetables	1.00 [Reference]	0.96 (0.74, 1.26)	0.85 (0.65, 1.12)	0.97 (0.74, 1.29)	0.722
Fruit	1.00 [Reference]	0.86 (0.66, 1.12)	0.88 (0.67, 1.14)	0.91 (0.69, 1.20)	0.565
Potatoes	1.00 [Reference]	1.18 (0.90, 1.55)	1.02 (0.78, 1.35)	1.23 (0.94, 1.61)	0.234
Dairy	1.00 [Reference]	1.07 (0.82, 1.39)	0.89 (0.67, 1.18)	0.99 (0.74, 1.31)	0.669
Legumes	1.00 [Reference]	0.96 (0.74, 1.24)	0.80 (0.61, 1.06)	0.93 (0.70, 1.23)	0.431
Soy	1.00 [Reference]	0.87 (0.67, 1.14)	0.92 (0.70, 1.20)	0.83 (0.63, 1.10)	0.262
Beef, Lamb, Pork	1.00 [Reference]	0.89 (0.68, 1.15)	0.92 (0.71, 1.20)	0.86 (0.65, 1.14)	0.359
Chicken	1.00 [Reference]	0.97 (0.74, 1.27)	0.94 (0.71, 1.23)	0.95 (0.72, 1.26)	0.699
Eggs	1.00 [Reference]	0.88 (0.67, 1.15)	0.98 (0.75, 1.28)	0.85 (0.64, 1.13)	0.381
Fish	1.00 [Reference]	0.91 (0.69, 1.18)	0.92 (0.70, 1.21)	0.90 (0.68, 1.20)	0.528
Nuts	1.00 [Reference]	0.92 (0.70, 1.20)	0.91 (0.69, 1.19)	0.91 (0.69, 1.21)	0.549
Sugars	1.00 [Reference]	0.89 (0.69, 1.17)	0.92 (0.70, 1.20)	0.87 (0.66, 1.16)	0.407
Fats ratio	1.00 [Reference]	0.86 (0.66, 1.12)	0.94 (0.72, 1.23)	0.87 (0.65, 1.15)	0.446

* Adjusted for: sex, age, educational level, smoking status, alcohol consumption, physical activity, energy intake, BMI, hypertension, and cholesterol levels.

Q1 = first quartile
Q2 = second quartile
Q3 = third quartile
Q4 = fourth quartile
HR = Hazard Ratios
95% CI = 95% Confidence Intervals
p-trend = p-value for trend
HRDea-score = Energy-adjusted Healthy Reference Diet score

Table S8. Hazard ratios (HRs) and 95% confidence intervals (CIs) for the association between quartiles of the HRDea-score and incidence of haemorrhagic stroke, excluding, one at a time, each component of the HRDea-score (*n*=35,496).

	HRDea-score				
	Q1 (32-66) (*n*=8874)	Q2 (67-73) (*n*=8874)	Q3 (74-79) (*n*=8874)	Q4 (80-117) (*n*=8874)	*P*-trend
HR (95% CI)					
HRDea-score recalculated excluding*:					
Whole Grains	1.00 [Reference]	1.26 (0.87, 1.81)	0.75 (0.49, 1.13)	0.93 (0.62, 1.40)	0.298
Vegetables	1.00 [Reference]	1.05 (0.73, 1.51)	0.77 (0.52, 1.15)	0.80 (0.53, 1.19)	0.142
Fruit	1.00 [Reference]	0,91 (0.63, 1.31)	0.83 (0.57, 1.21)	0.75 (0.50, 1.13)	0.147
Potatoes	1.00 [Reference]	0.88 (0.61, 1.27)	0.85 (0.59, 1.23)	0.75 (0.51, 1.10)	0.144
Dairy	1.00 [Reference]	1.03 (0.71, 1.51)	0.99 (0.67, 1.46)	0.78 (0.51, 1.18)	0.242
Legumes	1.00 [Reference]	0.82 (0.57, 1.18)	0.68 (0.46, 1.00)	0.76 (0.51, 1.13)	0.126
Soy	1.00 [Reference]	1.17 (0.82, 1.68)	0.63 (0.42, 0.96)	0.85 (0.57, 1.27)	0.129
Beef, Lamb, Pork	1.00 [Reference]	1.29 (0.90, 1.84)	0.69 (0.45, 1.05)	0.89 (0.59, 1.33)	0.160
Chicken	1.00 [Reference]	1.23 (0.85, 1.76)	0.66 (0.43, 1.01)	0.90 (0.60, 1.34)	0.196
Eggs	1.00 [Reference]	0.97 (0.67, 1.41)	0.80 (0.54, 1.18)	0.73 (0.48, 1.10)	0.085
Fish	1.00 [Reference]	1.27 (0.88, 1.81)	0.67 (0.44, 1.02)	0.92 (0.61, 1.37)	0.216
Nuts	1.00 [Reference]	1.25 (0.87, 1.79)	0.69 (0.45, 1.05)	0.92 (0.61, 1.38)	0.242
Sugars	1.00 [Reference]	1.27 (0.89, 1.82)	0.71 (0.47, 1.07)	0.89 (0.59, 1.34)	0.187
Fats ratio	1.00 [Reference]	1.16 (0.81, 1.67)	0.76 (0.51, 1.14)	0.83 (0.55, 1.25)	0.147

* Adjusted for: sex, age, educational level, smoking status, alcohol consumption, physical activity, energy intake, BMI, hypertension, and cholesterol levels.

Q1 = first quartile
Q2 = second quartile
Q3 = third quartile
Q4 = fourth quartile
HR = Hazard Ratios
95% CI = 95% Confidence Intervals
p-trend = p-value for trend
HRDea-score = Energy-adjusted Healthy Reference Diet score

Table S9. Hazard ratios (HRs) and 95% confidence intervals (CIs) for the association between quartiles of the HRDea-score and incidence of CVD, CHD, total stroke, ischaemic and haemorrhagic stroke, applying IPTWs (*n*=35,496).

	Quartiles of HRDea-score (range)				
	Q1 (32-66) (*n*=8874)	Q2 (67-73) (*n*=8874)	Q3 (74-79) (*n*=8874)	Q4 (80-117) (*n*=8874)	*P*-trend
CVD, *n*	1100	1140	979	934	
Persons-years	122061	125076	127678	136736	
Unadjusted Model	1.00 [ref]	1.00 (0.91 – 1.09)	0.83 (0.75 – 0.91)	0.78 (0.69 – 0.89)	<0.001
Model 1*	1.00 [ref]	0.99 (0.90 – 1.09)	0.83 (0.76 – 0.92)	0.80 (0.71 – 0.91)	<0.001
Model 2†	1.00 [ref]	1.00 (0.91 – 1.09)	0.84 (0.76 – 0.92)	0.83 (0.74 – 0.93)	<0.001
Model 3‡	1.00 [ref]	1.00 (0.91 – 1.10)	0.83 (0.75 – 0.92)	0.82 (0.73 – 0.92)	<0.001
CHD, *n*	617	614	542	544	
Persons-years	124454	127252	129333	138622	
Unadjusted Model	1.00 [ref]	1.00 (0.88 – 1.13)	0.88 (0.77 – 1.00)	0.83 (0.71 – 0.98)	0.007
Model 1*	1.00 [ref]	1.00 (0.88 – 1.13)	0.89 (0.78 – 1.01)	0.85 (0.72 – 0.99)	0.016
Model 2†	1.00 [ref]	1.01 (0.89 – 1.14)	0.89 (0.78 – 1.02)	0.87 (0.75 – 1.02)	0.033
Model 3‡	1.00 [ref]	1.00 (0.89 – 1.13)	0.88 (0.77 – 1.00)	0.86 (0.74 – 1.00)	0.014
Total Stroke, *n*	197	233	203	205	
Persons-years	127190	129916	131765	141150	
Unadjusted Model	1.00 [ref]	1.15 (0.93 – 1.42)	0.89 (0.72 - 1.11)	0.94 (0.69 – 1.27)	0.380
Model 1*	1.00 [ref]	1.14 (0.93 – 1.41)	0.90 (0.73 - 1.12)	0.98 (0.72 – 1.32)	0.570
Model 2†	1.00 [ref]	1.14 (0.93 – 1.41)	0.92 (0.74 – 1.14)	1.00 (0.75 – 1.34)	0.692
Model 3‡	1.00 [ref]	1.15 (0.93 – 1.42)	0.91 (0.73 – 1.14)	0.99 (0.74 – 1.32)	0.618
Ischaemic stroke, *n*	112	118	125	123	
Persons-years	127545	130323	131980	141489	
Unadjusted Model	1.00 [ref]	1.07 (0.80 – 1.42)	0.99 (0.75 - 1.31)	1.04 (0.66 – 1.61)	0.951
Model 1*	1.00 [ref]	1.06 (0.80 – 1.41)	1.00 (0.76 - 1.33)	1.08 (0.70 – 1.67)	0.779
Model 2†	1.00 [ref]	1.06 (0.80 – 1.41)	1.03 (0.77 – 1.37)	1.10 (0.74 – 1.65)	0.673
Model 3‡	1.00 [ref]	1.06 (0.80 – 1.42)	1.02 (0.76 – 1.35)	1.08 (0.73 – 1.60)	0.750
Haemorrhagic stroke, *n*	54	76	45	58	
Persons-years	127921	130593	132462	141839	
Unadjusted Model	1.00 [ref]	1.28 (0.88 – 1.88)	0.68 (0.44 - 1.05)	0.90 (0.57 – 1.40)	0.222
Model 1*	1.00 [ref]	1.27 (0.87 – 1.86)	0.68 (0.45 - 1.05)	0.94 (0.60 – 1.45)	0.310
Model 2†	1.00 [ref]	1.28 (0.88 – 1.88)	0.70 (0.46 – 1.08)	0.97 (0.62 – 1.52)	0.414
Model 3‡	1.00 [ref]	1.29 (0.88 – 1.88)	0.70 (0.46 – 1.08)	0.97 (0.62 – 1.53)	0.422

*Adjusted for age and sex.
†Adjusted for age, sex, educational level, smoking status, alcohol consumption, physical activity, and energy intake.
‡Adjusted for age, sex, educational level, smoking status, alcohol consumption, physical activity, energy intake, BMI, hypertension, and total cholesterol levels.

Q1 = first quartile
Q2 = second quartile
Q3 = third quartile
Q4 = fourth quartile

HRs = Hazard Ratios
95% CI = 95% Confidence Intervals
p-trend = p-value for trend
HRDea-score = Energy-adjusted Healthy Reference Diet score
IPTWs = Inverse probability of treatment weights
CVD = cardiovascular disease
CHD = coronary heart disease

Table S10. Hazard ratios (HRs) and 95% confidence intervals (CIs) for the association between quartiles of the mMDS and incidence of CVD, CHD, total stroke, ischaemic and haemorrhagic stroke (*n*=35,496).

	Quartiles of mMED (range)				
	Q1 (0 - 3) (*n*=10,738)	Q2 (4) (*n*=8,160)	Q3 (5) (*n*=7,910)	Q4 (6-9) (*n*=8,688)	*P*-trend
CVD, *n*	1466	979	869	839	
Persons-years	152651	116652	113355	124945	
Unadjusted Model	1.00 [ref]	0.87 (0.80 – 0.95)	0.80 (0.73 – 0.87)	0.70 (0.64 – 0.76)	<0.001
Model 1*	1.00 [ref]	0.87 (0.80 – 0.94)	0.82 (0.75 – 0.89)	0.74 (0.68 – 0.80)	<0.001
Model 2†	1.00 [ref]	0.91 (0.84 – 0.98)	0.89 (0.82 – 0.97)	0.82 (0.75 – 0.90)	<0.001
Model 3‡	1.00 [ref]	0.91 (0.83 – 0.98)	0.91 (0.83 – 0.99)	0.84 (0.77 – 0.92)	<0.001
CHD, *n*	822	564	489	480	
Persons-years	155675	118574	115141	126718	
Unadjusted Model	1.00 [ref]	0.90 (0.81 – 1.00)	0.80 (0.72 – 0.90)	0.72 (0.64 – 0.80)	<0.001
Model 1*	1.00 [ref]	0.89 (0.80 – 0.99)	0.82 (0.73 – 0.92)	0.74 (0.66 – 0.83)	<0.001
Model 2†	1.00 [ref]	0.94 (0.84 – 1.04)	0.89 (0.80 – 1.00)	0.84 (0.75 – 0.94)	0.002
Model 3‡	1.00 [ref]	0.94 (0.84 – 1.05)	0.91 (0.81 – 1.02)	0.86 (0.76 – 0.96)	0.008
Total Stroke, *n*	308	179	173	178	
Persons-years	159229	121253	117295	128806	
Unadjusted Model	1.00 [ref]	0.76 (0.63 – 0.92)	0.76 (0.63 – 0.92)	0.72 (0.60 – 0.86)	<0.001
Model 1*	1.00 [ref]	0.77 (0.64 – 0.93)	0.81 (0.67 – 0.98)	0.79 (0.66 – 0.95)	0.013
Model 2†	1.00 [ref]	0.80 (0.67 – 0.97)	0.88 (0.73 – 1.06)	0.88 (0.73 – 1.07)	0.225
Model 3‡	1.00 [ref]	0.80 (0.66 – 0.96)	0.88 (0.73 – 1.07)	0.89 (0.74 – 1.08)	0.264
Ischaemic stroke, *n*	180	99	88	111	
Persons-years	159652	121545	117689	129088	
Unadjusted Model	1.00 [ref]	0.72 (0.57 – 0.92)	0.66 (0.51 – 0.86)	0.76 (0.60 – 0.97)	0.010
Model 1*	1.00 [ref]	0.73 (0.57 – 0.94)	0.71 (0.55 – 0.91)	0.85 (0.67 – 1.08)	0.099
Model 2†	1.00 [ref]	0.76 (0.60 – 0.98)	0.78 (0.60 – 1.01)	0.96 (0.76 – 1.23)	0.550
Model 3‡	1.00 [ref]	0.77 (0.60 – 0.99)	0.78 (0.60 – 1.01)	0.99 (0.77 – 1.26)	0.662
Haemorrhagic stroke, *n*	86	53	56	38	
Persons-years	1680423	121878	117855	129472	
Unadjusted Model	1.00 [ref]	0.81 (0.58 – 1.14)	0.89 (0.63 – 1.24)	0.55 (0.38 – 0.80)	0.006
Model 1*	1.00 [ref]	0.83 (0.59 – 1.16)	0.94 (0.67 – 1.31)	0.60 (0.41 – 0.88)	0.025
Model 2†	1.00 [ref]	0.84 (0.60 – 1.19)	0.98 (0.70 – 1.38)	0.63 (0.43 – 0.93)	0.060
Model 3‡	1.00 [ref]	0.80 (0.57 – 1.14)	0.97 (0.69 – 1.37)	0.63 (0.42 – 0.93)	0.060

*Adjusted for age and sex.
†Adjusted for age, sex, educational level, smoking status, alcohol consumption, physical activity, and energy intake.
‡Adjusted for age, sex, educational level, smoking status, alcohol consumption, physical activity, energy intake, BMI, hypertension, and total cholesterol levels.

Q1 = first quartile
Q2 = second quartile
Q3 = third quartile
Q4 = fourth quartile
HRs = Hazard Ratios
95% CI = 95% Confidence Intervals
p-trend = p-value for trend
mMED = modified Mediterranean Diet
CVD = cardiovascular disease
CHD = coronary heart disease

Table S11. Hazard ratios (HRs) and 95% confidence intervals (CIs) for the association between tertials of the HDI and incidence of CVD, CHD, total stroke, ischaemic and haemorrhagic stroke (*n*=35,496).

	Tertiles of HDI			
	Q1 (1 - 3) (*n*=7,354)	Q2 (4) (*n*=7,560)	Q3 (5 -7) (*n*=4,964)	*P*-trend
CVD, *n*	993	892	605	
Persons-years	103818	108461	71169	
Unadjusted Model	1.00 [ref]	0.86 (0.78 – 0.94)	0.89 (0.80 – 0.98)	0.008
Model 1*	1.00 [ref]	0.84 (0.76 – 0.91)	0.80 (0.72 – 0.88)	<0.001
Model 2†	1.00 [ref]	0.88 (0.80 – 0.96)	0.91 (0.82 – 1.01)	0.036
Model 3‡	1.00 [ref]	0.88 (0.81 – 0.97)	0.92 (0.83 – 1.02)	0.067
CHD, *n*	569	492	329	
Persons-years	105842	110218	72303	
Unadjusted Model	1.00 [ref]	0.83 (0.73 – 0.93)	0.84 (0.74 – 0.97)	0.006
Model 1*	1.00 [ref]	0.81 (0.72 – 0.92)	0.78 (0.68 – 0.90)	<0.001
Model 2†	1.00 [ref]	0.86 (0.76 – 0.97)	0.88 (0.77 – 1.01)	0.040
Model 3‡	1.00 [ref]	0.86 (0.76 – 0.97)	0.90 (0.78 – 1.03)	0.073
Total Stroke, *n*	183	205	115	
Persons-years	108548	112241	73867	
Unadjusted Model	1.00 [ref]	1.08 (0.88 – 1.32)	0.92 (0.73 – 1.16)	0.601
Model 1*	1.00 [ref]	1.03 (0.85 – 1.26)	0.78 (0.61 – 0.98)	0.050
Model 2†	1.00 [ref]	1.08 (0.88 – 1.32)	0.89 (0.70 – 1.12)	0.423
Model 3‡	1.00 [ref]	1.07 (0.88 – 1.32)	0.89 (0.70 – 1.13)	0.430
Ischaemic stroke, *n*	113	104	73	
Persons-years	108806	112574	74017	
Unadjusted Model	1.00 [ref]	0.89 (0.68 – 1.16)	0.95 (0.71 – 1.27)	0.651
Model 1*	1.00 [ref]	0.84 (0.65 – 1.10)	0.79 (0.59 – 1.06)	0.101
Model 2†	1.00 [ref]	0.88 (0.67 – 1.15)	0.92 (0.68 – 1.24)	0.513
Model 3‡	1.00 [ref]	0.88 (0.67 – 1.16)	0.95 (0.70 – 1.29)	0.663
Haemorrhagic stroke, *n*	42	63	32	
Persons-years	109287	112859	74241	
Unadjusted Model	1.00 [ref]	1.45 (0.98 – 2.14)	1.12 (0.71 – 1.77)	0.502
Model 1*	1.00 [ref]	1.39 (0.94 – 2.06)	0.95 (0.60 – 1.51)	0.970
Model 2†	1.00 [ref]	1.46 (0.99 - 2.16)	0.99 (0.62 – 1.59)	0.856
Model 3‡	1.00 [ref]	1.41 (0.95 – 2.10)	0.92 (0.56 – 1.49)	0.898

*Adjusted for age and sex.

†Adjusted for age, sex, educational level, smoking status, alcohol consumption, physical activity, and energy intake.

‡Adjusted for age, sex, educational level, smoking status, alcohol consumption, physical activity, energy intake, BMI, hypertension, and total cholesterol levels.

Q1 = first quartile
Q2 = second quartile
Q3 = third quartile
Q4 = fourth quartile
HRs = Hazard Ratios
95% CI = 95% Confidence Intervals
p-trend = p-value for trend
HDI = Healthy Diet Indicator
CVD = cardiovascular disease
CHD = coronary heart disease

Table S12. Hazard ratios (HRs) and 95% confidence intervals (CIs) for the association between PDI and incidence of CVD, CHD, total stroke, ischaemic and haemorrhagic stroke (*n*=35,496).

	PDI (26 - 73) (*n*=35,496)	95% (CI)	P-value
CVD, *n*	2,299		
Persons-years	264797		
Unadjusted Model	0.99	(0.98 – 0.99)	<0.001
Model 1*	0.99	(0.99 – 1.00)	0.001
Model 2†	0.99	(0.99 – 1.00)	0.039
Model 3‡	1.00	(0.99 – 1.00)	0.404
CHD, *n*	1,309		
Persons-years	269418		
Unadjusted Model	0.99	(0.98 – 1.00)	0.002
Model 1*	0.99	(0.99 – 1.00)	0.047
Model 2†	0.99	(0.99 – 1.00)	0.070
Model 3‡	1.00	(0.99 – 1.00)	0.350
Total Stroke, *n*	461		
Persons-years	275323		
Unadjusted Model	0.99	(0.98 – 1.00)	0.043
Model 1*	0.99	(0.98 – 1.00)	0.169
Model 2†	1.00	(0.99 – 1.01)	0.675
Model 3‡	1.00	(0.99 – 1.01)	0.939
Ischaemic stroke, *n*	259		
Persons-years	276059		
Unadjusted Model	0.99	(0.98 – 1.01)	0.495
Model 1*	1.00	(0.98 – 1.01)	0.837
Model 2†	1.01	(0.99 – 1.02)	0.489
Model 3‡	1.01	(0.99 – 1.02)	0.284
Haemorrhagic stroke, *n*	132		
Persons-years	276958		
Unadjusted Model	0.98	(0.95 – 1.00)	0.028
Model 1*	0.98	(0.96 – 1.00)	0.057
Model 2†	0.99	(0.96 – 1.01)	0.212
Model 3‡	0.99	(0.97 – 1.01)	0.413

*Adjusted for age and sex.
†Adjusted for age, sex, educational level, smoking status, alcohol consumption, physical activity, and energy intake.
‡Adjusted for age, sex, educational level, smoking status, alcohol consumption, physical activity, energy intake, BMI, hypertension, and total cholesterol levels.

HRs = Hazard Ratios
95% CI = 95% Confidence Intervals
PDI = Plant-based Diet Index
CVD = cardiovascular disease
CHD = coronary heart disease

Table S13. Correlation coefficients for the HRDea with mMED, HDI, and PDI.

	Correlation Coefficient*
HRDea x mMED	r = 0.38
HRDea x HDI	r = 0.23
HRDea x PDI	ρ = 0.35

*Calculated using either Pearson's correlation coefficient (r), or Spearman's rank correlation (ρ)

HRDea = Energy-adjusted Healthy Reference Diet

mMED = modified Mediterranean Diet

HDI = Healthy Diet Indicator

PDI = Plant-based Diet Index

Table S14. Percent difference for the association between quartiles of the HRDea-score and environmental indicators, adjusted for age, sex, and energy intake (n=35,496).

	Q4 vs Q1*	95% CI
GHGE (kg CO2-eq)	-2.4	(-5.0, 0.2)
Land Use (m^2/y)	-3.9	(-5.2, -2.6)
Blue water use (m^3/d)	32.1	(28.5, 35.7)
Freshwater eutrophication (Kg P-eq)	-0.5	(-2.6, 1.6)
Marine eutrophication (Kg N-eq)	-3.3	(-5.8, -0.8)
Terrestrial acidification (Kg SO2-eq)	-7.7	(-10.8, -4.6)

* Expressed in %

Q1 = first quartile
Q4 = fourth quartile
Kg CO_2-eq = kilograms of carbon dioxide equivalent
m^2 per year = square meters per year
m^3 per day = cubic meters per year
Kg P-eq = kilograms of phosphorus equivalent
Kg N-eq = kilograms of nitrogen equivalent
Kg SO2-eq = kilograms of sulphur dioxide equivalent

Figure S1. Flowchart of the study population.

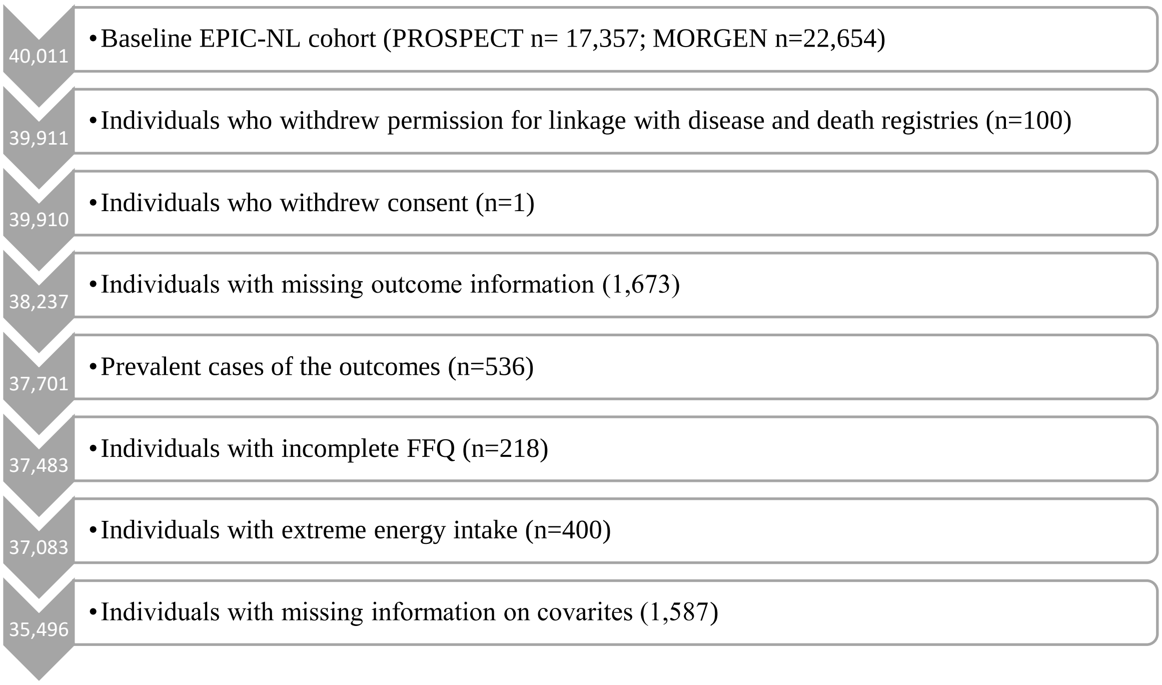

Figure S2. Hazard ratios (HRs) and 95% confidence intervals (CIs) for the association between HRDea-score and CVD, stratified by sex, age, BMI, and educational level.

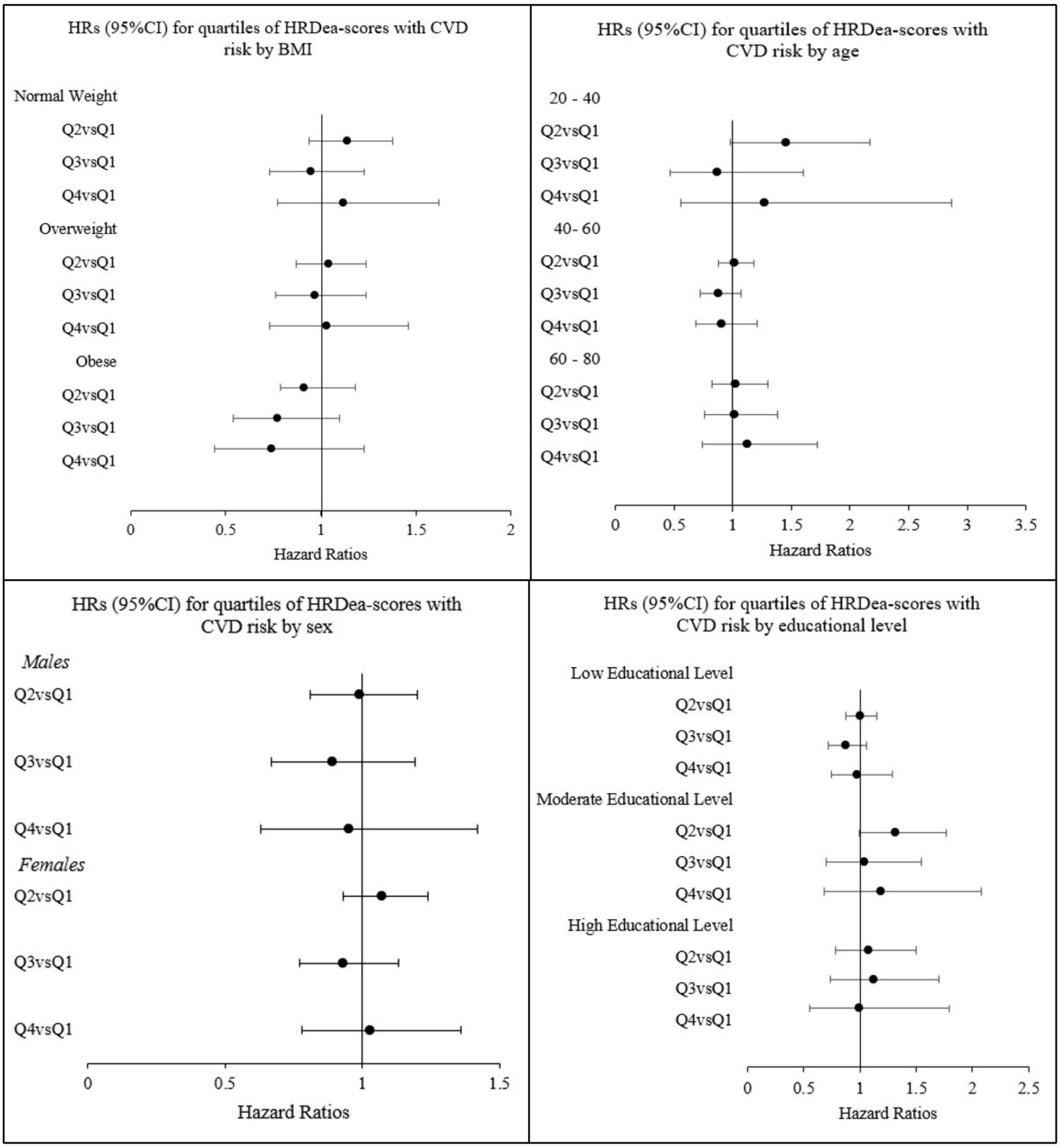

HRs = Hazard Ratios
95% CI = 95% confidence intervals
HRDea-score = Energy-adjusted Healthy Reference Diet score
CVD = cardiovascular disease
BMI = Body Mass Index

Figure S3. Hazard ratios (HRs) and 95% confidence intervals (CIs) for the association between HRDea-score and CHD, stratified by sex, age, BMI, and educational level.

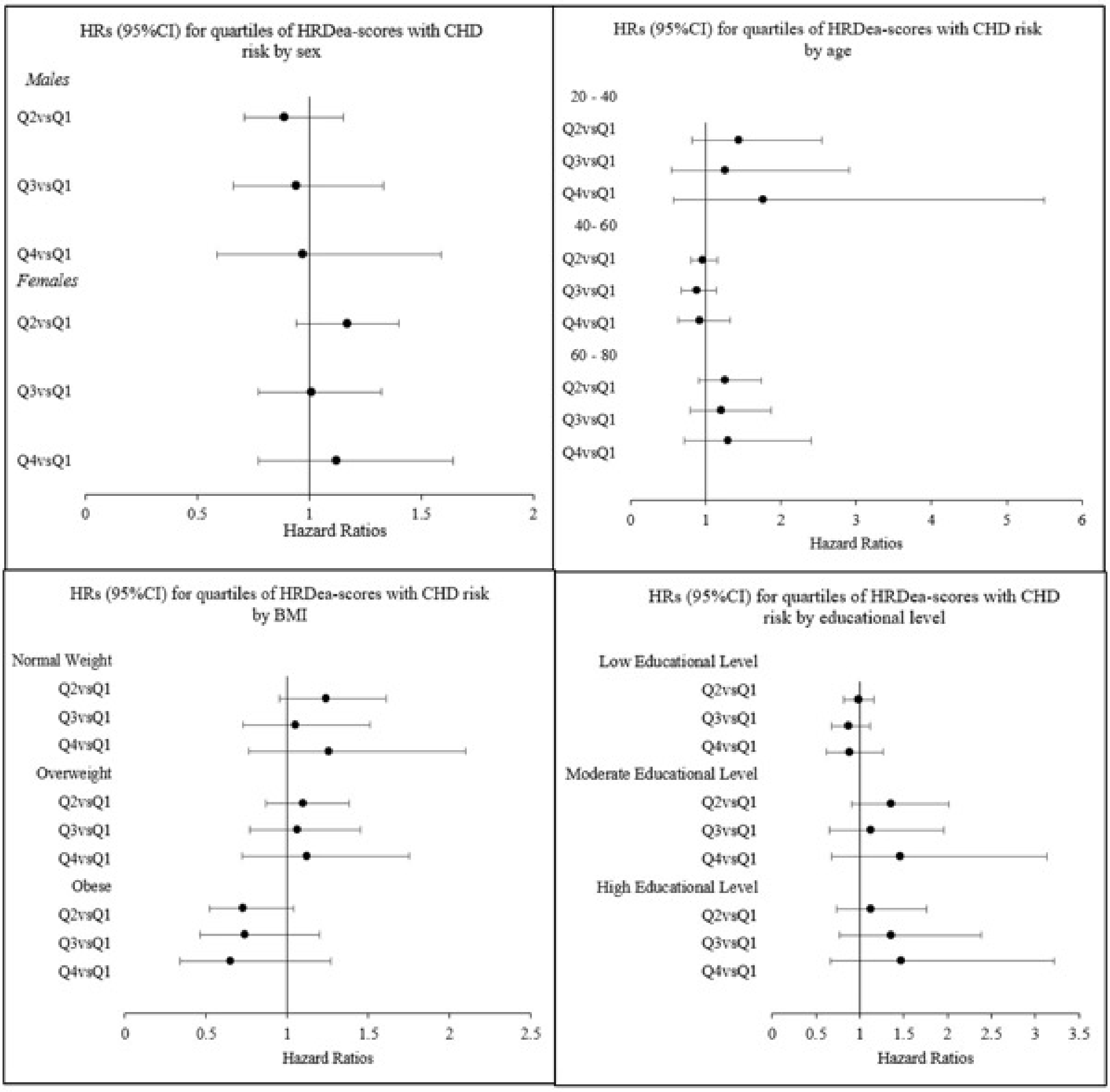

HRs = Hazard Ratios
95% CI = 95% confidence intervals
HRDea-score = Energy-adjusted Healthy Reference Diet score
CHD = coronary heart disease
BMI = Body Mass Index

Figure S4. Hazard ratios (HRs) and 95% confidence intervals (CIs) for the association between HRDea-score and total stroke, stratified by sex, age, BMI, and educational level.

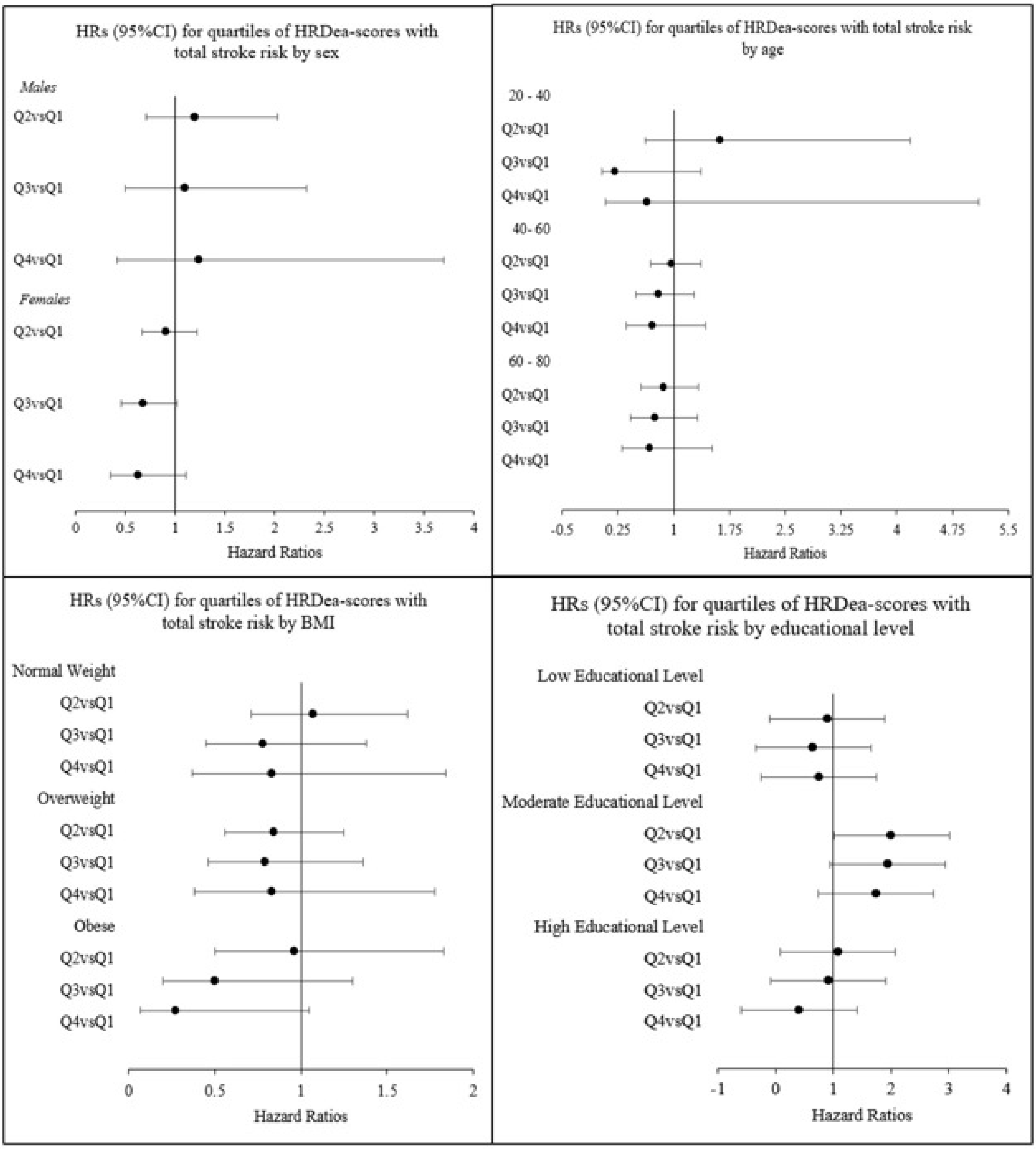

HRs = Hazard Ratios
95% CI = 95% confidence intervals
HRDea-score = Energy-adjusted Healthy Reference Diet score
BMI = Body Mass Index

Figure S5. Hazard ratios (HRs) and 95% confidence intervals (CIs) for the association between HRDea-score and ischemic stroke, stratified by sex, age, BMI, and educational level.

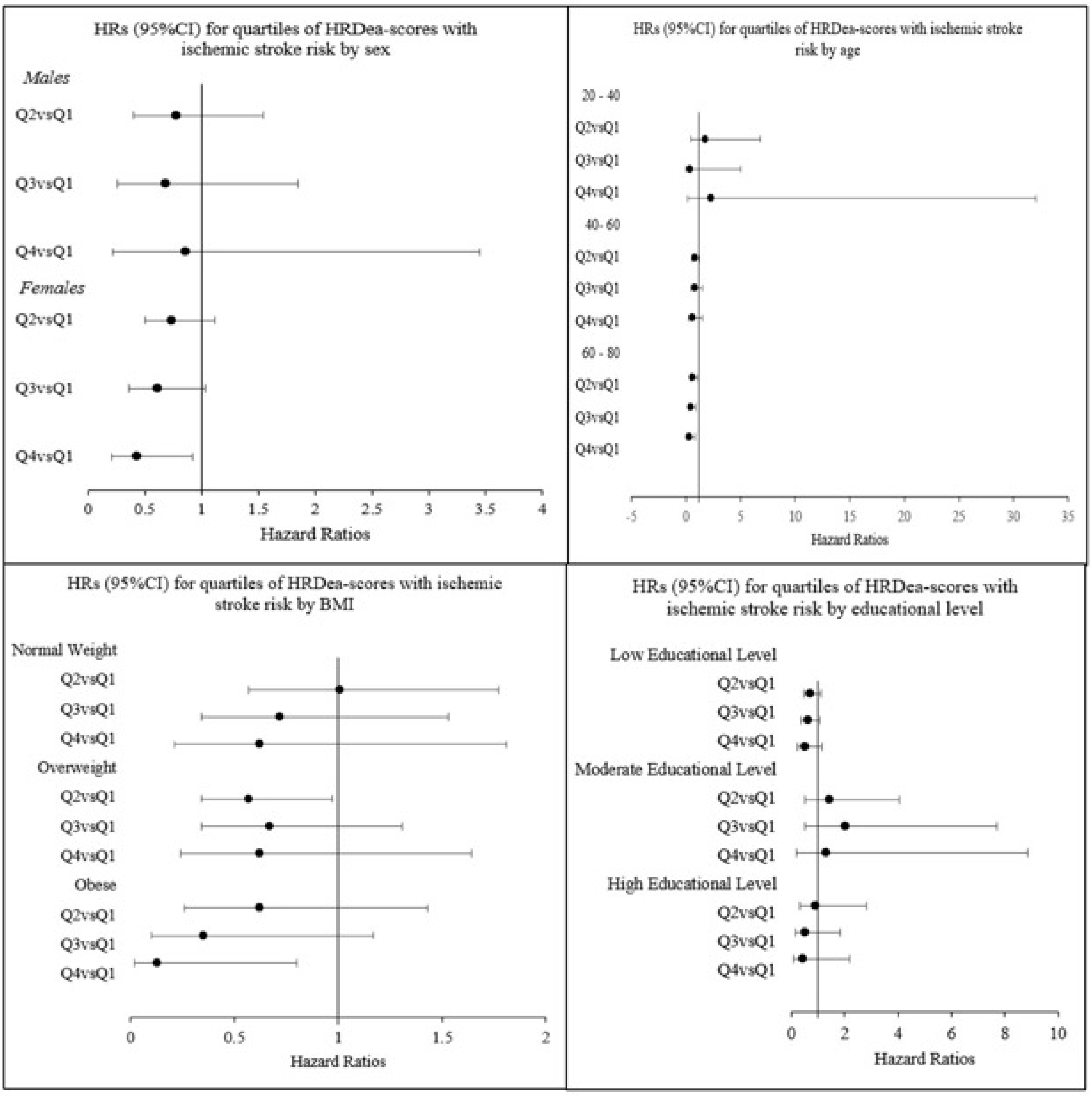

HRs = Hazard Ratios
95% CI = 95% confidence intervals
HRDea-score = Energy-adjusted Healthy Reference Diet score
BMI = Body Mass Index

Figure S6. Hazard ratios (HRs) and 95% confidence intervals (CIs) for the association between HRDea-score and haemorrhagic stroke, stratified by sex, age, BMI, and educational level.

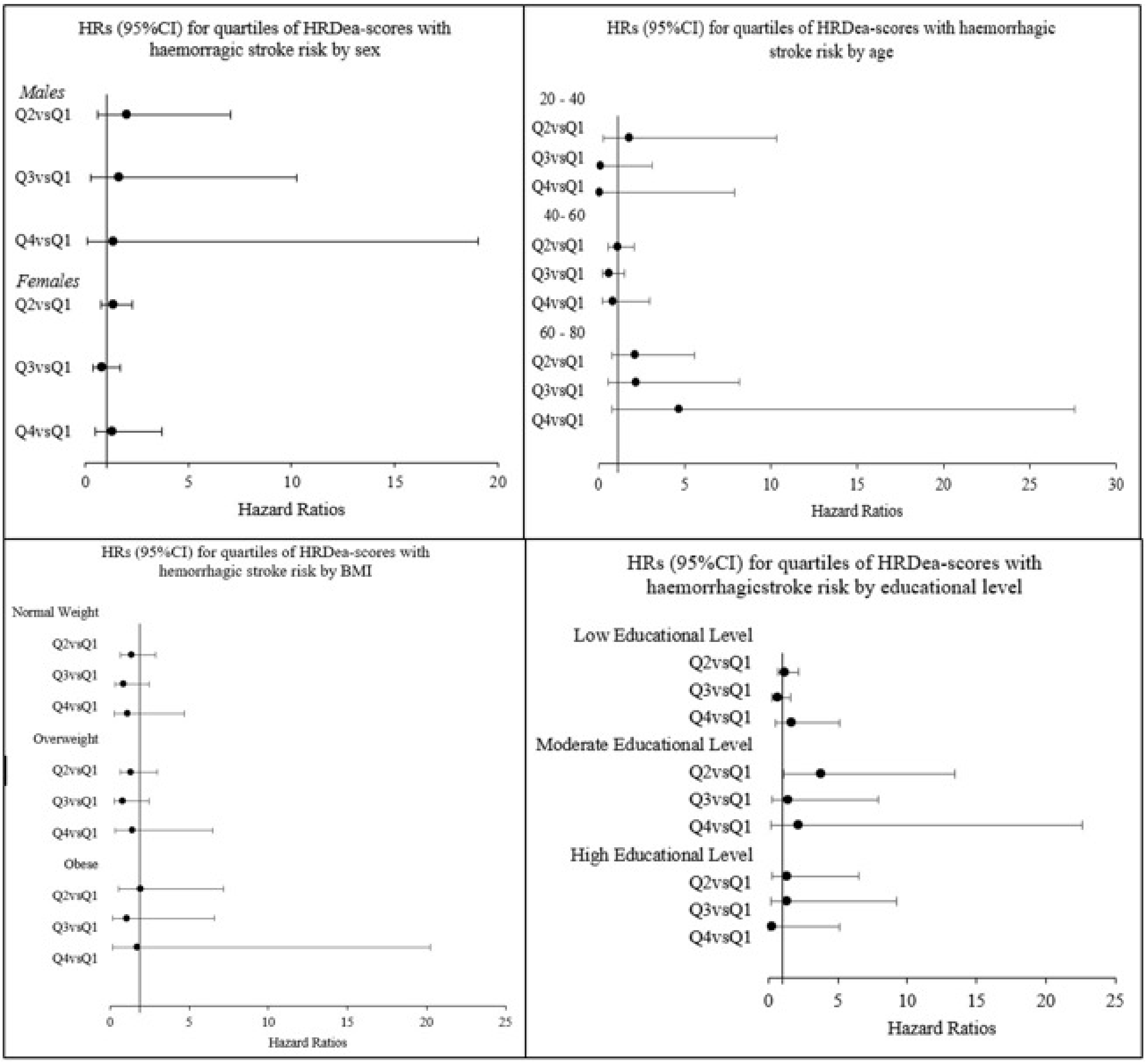

HRs = Hazard Ratios
95% CI = 95% confidence intervals
HRDea-score = Energy-adjusted Healthy Reference Diet score
BMI = Body Mass Index

Figure S7. Hazard ratios (HRs) and 95% confidence intervals (CIs) for the association between HRDea-score and CVD, CHD, total stroke, ischemic stroke, and haemorrhagic stroke, using restricted cubic splines.

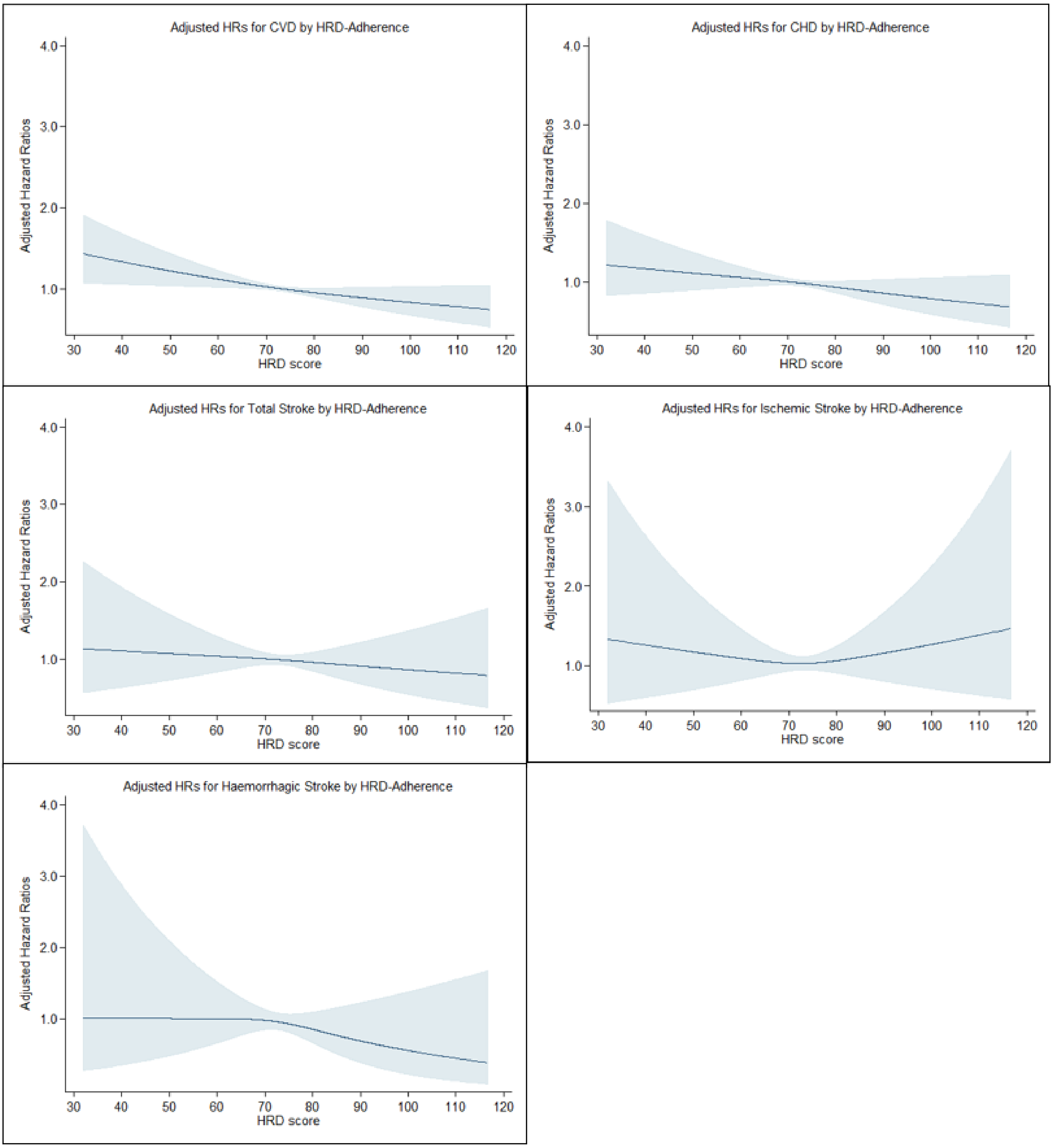

95% CI = 95% confidence intervals
HRDea-score = Energy-adjusted Healthy Reference Diet score
CVD = cardiovascular disease
CHD = coronary heart disease

Figure S8. Regression coefficients and 95% CI for the associations between HRDea-score and greenhouse gas emissions, land use, blue water use, freshwater use, marine eutrophication, and terrestrial acidification, using restricted cubic splines.

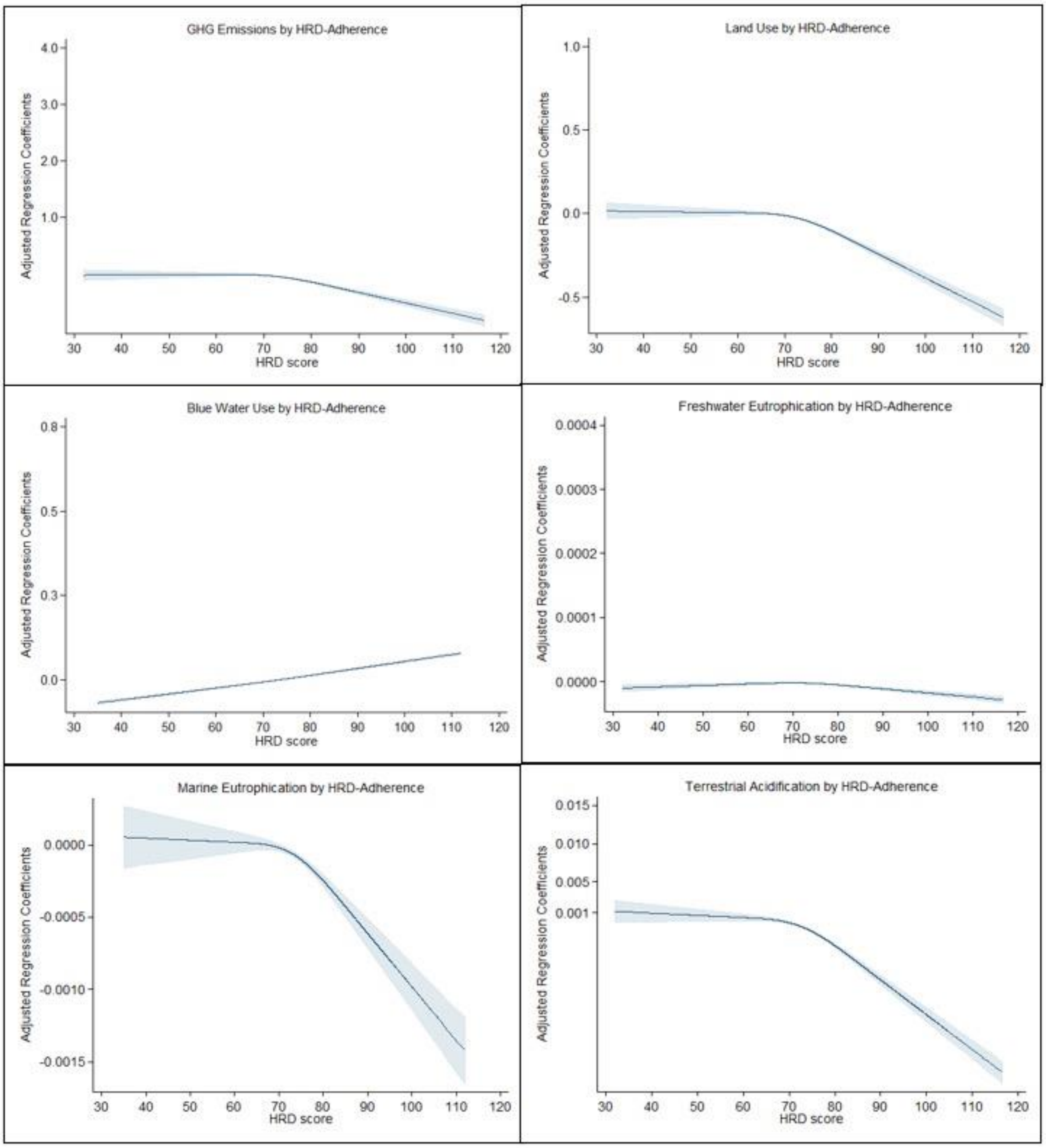

95% CI = 95% confidence intervals
HRDea-score = Energy-adjusted Healthy Reference Diet score
GHG = Greenhouse Gas
